# Shear stress-stimulated AMPK couples endothelial cell mechanics, metabolism and vasodilation

**DOI:** 10.1242/jcs.262232

**Published:** 2024-12-18

**Authors:** Nicholas M. Cronin, Logan W. Dawson, Kris A. DeMali

**Affiliations:** Roy J. and Lucille A. Carver College of Medicine at the University of Iowa, Department of Biochemistry and Molecular Biology, 51 Newton Rd, Iowa City, IA 52242, USA

**Keywords:** Cell–cell adhesion, Mechanotransduction, VE-cadherin

## Abstract

Endothelial cells respond to mechanical force by stimulating cellular signaling, but how these pathways are linked to elevations in cell metabolism and whether metabolism supports the mechanical response remains poorly understood. Here, we show that the application of force to endothelial cells stimulates VE-cadherin to activate liver kinase B1 (LKB1; also known as STK11) and AMP-activated protein kinase (AMPK), a master regulator of energy homeostasis. VE-cadherin-stimulated AMPK increases eNOS (also known as NOS3) activity and localization to the plasma membrane, reinforcement of the actin cytoskeleton and cadherin adhesion complex, and glucose uptake. We present evidence for the increase in metabolism being necessary to fortify the adhesion complex, actin cytoskeleton and cellular alignment. Together, these data extend the paradigm for how mechanotransduction and metabolism are linked to include a connection to vasodilation, thereby providing new insight into how diseases involving contractile, metabolic and vasodilatory disturbances arise.

## Teaser

**Highlighted Article:** The endothelial cadherin complex mediates AMPK activation through recruitment of LKB1 to support actin cytoskeletal dynamics, increased glycolysis and vasodilation via increased eNOS activity.

## INTRODUCTION

All cells in the human body experience mechanical forces. These forces are sensed by cell surface receptors and are propagated to the cell interior. To counteract these forces, cells reinforce their adhesion to neighboring cells and to their actin cytoskeletons (Liu et al., 2010; Chanet and Martin, 2014). Reinforcement requires increases in protein synthesis, transport, actomyosin contractility and actin polymerization – all of which are processes that require energy (Daniel et al., 1986; Bernstein and Bamburg, 2003).

How cells derive the energy needed to support reinforcement of cell–cell adhesions and the actin cytoskeleton is an area of active investigation. In response to increases in tension, modifications to the enzymes and allosteric modulators involved in energy metabolism are altered (Dawson et al., 2023). In epithelial cells, E-cadherin senses external forces, and the tension is propagated through the protein to produce conformational changes on the cytoplasmic domain (Borghi et al., 2012). These conformational changes are believed to allow for the recruitment of binding partners and the activation of signal transduction cascades. One such signal transduction cascade is initiated by AMP-activated protein kinase (AMPK), a master regulator of cell metabolism. AMPK is activated and recruited to E-cadherin in response to the application of external forces (Bays et al., 2014). Active AMPK has two effects. It stimulates a signal transduction cascade that culminates in robust changes in the actin cytoskeleton allowing the cell to resist increased external tension. Additionally, it increases glucose uptake through glucose transporter 1 (GLUT1; also known as SLC2A1), which is required for the generation of ATP (Salvi et al., 2021). The increased ATP fuels the alterations in protein synthesis, transport, actomyosin contractility and actin polymerization needed for the cell to counter the applied tension.

Much of the work to date has been performed in epithelial cells. The broader applicability of this paradigm has yet to be rigorously tested. Endothelial cells offer a promising model for testing this possibility, as they share a common feature with epithelial cells: both express a force-sensing cell surface cadherin, namely vascular endothelial cadherin (VE-cadherin) in endothelial cells (Barry et al., 2015; Conway and Schwartz, 2015). Additionally, AMPK is activated in endothelial cells in response to force (Li et al., 2019). However, there are additional levels of complexity in endothelial cells that cast doubt as to whether mechanics and metabolism are coupled in the same manner as observed in epithelial cells. Key among these is that force sensing is more intricate in endothelial cells. VE-cadherin is a component of an adherens junction mechanosensory complex, including platelet endothelial cell adhesion molecule 1 (PECAM-1) and vascular endothelial growth factor receptor 2 (VEGFR2). Additionally, VE-cadherin only bears force when present in adherens junctions (Conway et al., 2013). This contrasts with E-cadherin, which bears force when it is on the cell surface and irrespective of its presence in adherens junctions (Borghi et al., 2012). Finally, although both endothelial and epithelial cells heavily rely upon glycolysis to meet their energetic demands, endothelial cells have fewer mitochondria and do not employ oxidative phosphorylation to the same extent as epithelial cells to generate energy (Dawson et al., 2023). These observations raise the question as to how AMPK is activated in response to external forces in endothelial and the consequences of its activation.

Here, we present evidence that the application of shear stress to endothelial cells stimulates a VE-cadherin dependent signal transduction cascade whereby liver kinase β1 (LKB1; also known as STK11) activates AMPK. Active AMPK phosphorylates endothelial nitric oxide synthase (eNOS; also known as NOS3), thereby coupling VE-cadherin stimulated mechanotransduction and vasodilation. VE-cadherin-stimulated AMPK signals for increased glucose uptake and metabolism, to provide the energy necessary for reinforcement of the actin cytoskeleton and cadherin adhesion complex and endothelial cell alignment. Taken together, this work extends the paradigm for how mechanotransduction and metabolism are linked to include a connection to vasodilation.

## RESULTS

### The cadherin adhesion complex is required for shear stress-induced activation of AMPK

Cells respond to mechanical cues from their environment by reinforcing their cytoskeleton and adhesions, a process that consumes considerable energy (Daniel et al., 1986; Bernstein and Bamburg, 2003; Choi and Helmke, 2008). The mechanisms by which this energy is produced remain poorly understood. However, in epithelial and endothelial cells, AMPK, a master regulator of cell metabolism, is activated in response to fluid shear stress (Young et al., 2009; Bays et al., 2017), suggesting its involvement. To explore the role of AMPK, we monitored its activation in human umbilical vein endothelial cells (HUVECs) and bovine aortic endothelial cells (BAECs) subjected to various durations and intensities of shear stress. Activation of AMPK was assessed by examining phosphorylation of AMPK in its activation loop using a Thr172 phospho-specific antibody, an approach that we and other groups have shown mirrors AMPK kinase activity and phosphorylation of its substrates (Bays et al., 2017). Consistent with previous reports, AMPK was activated in response to shear stress (Young et al., 2009). Maximal activation occurred with the application of 12 dynes/cm^2^ of shear stress and after 5 min in HUVECs (Fig. S1A,B) and in BAECs (Fig. S1D,E).

**Fig. 1.**
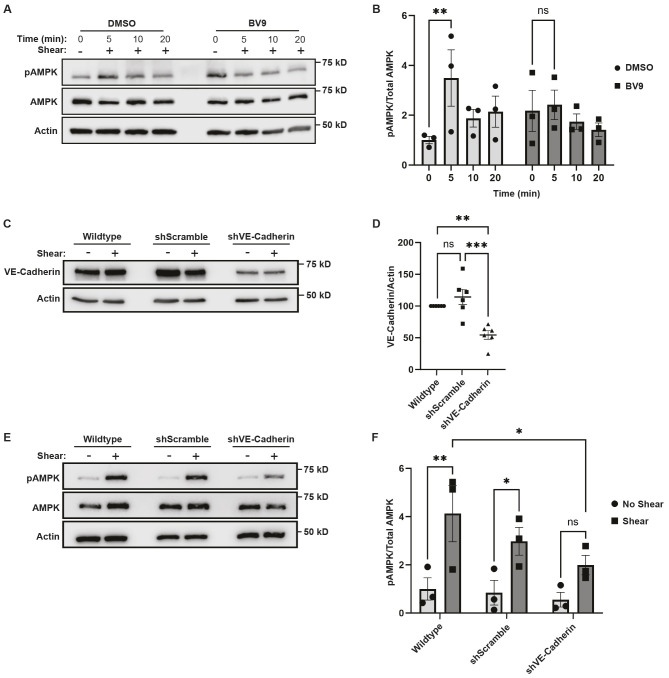
**VE-cadherin is required for shear-induced AMPK activation.** (A,B) Shear stimulated AMPK activation in the presence of VE-cadherin function-blocking antibodies. HUVECs were left resting (−) or exposed to shear stress (+) for the indicated times in the presence or absence of BV9, a VE-cadherin function-blocking antibody, or DMSO as a control. AMPK activation was examined by immunoblotting total cell lysates with an antibody that recognizes AMPK phosphorylated in its activation loop (pAMPK) or actin as a loading control. The blots were stripped and probed with antibodies that report on total AMPK (AMPK). The graph in B depicts the ratios of phosphorylated AMPK to total AMPK; the data are mean±s.e.m., *n*=3 biologically independent samples. (C,D) VE-cadherin inhibition. VE-cadherin expression was inhibited by treating HUVECs with lentiviruses encoding shRNAs targeting human VE-cadherin (shVE-Cadherin) or a scrambled sequence (shScramble). Wild-type parental cells were employed as a control. Total cell lysates were immunoblotted with antibodies against VE-cadherin to show the level of inhibition or actin as a loading control. Representative western blot images are shown C. The graphs in D depict the level of VE-cadherin normalized to total protein levels. The data are mean±s.e.m of an *n*=6 biologically independent samples. (E,F) Shear stress-induced AMPK activation in cells with depleted VE-cadherin levels. AMPK activation was examined as described above in A and B in wild-type, shScramble, or shVE-cadherin cells after 5 min of applied shear stress. The data are mean±s.e.m of an *n*=3 biologically independent samples. *P<0.05; **P<0.01; ***P<0.001; ns, not significant [two-way ANOVA with Tukey's comparison (B,D); one-way ANOVA with Dunnett's test (F)].

The activation of AMPK in response to shear stress suggests that components of the adherens junction complex might be necessary for this process. Previous work has demonstrated that VE-cadherin acts as an adaptor to elicit the function of the complex. To examine the role of VE-cadherin in shear stress-stimulated AMPK activation, cells were incubated with BV9, a VE-cadherin function-blocking antibody, or DMSO as a control. After treatment, cells were either left undisturbed (−) or subjected to 12 dynes/cm^2^ of shear stress (+) for durations ranging from 5 to 20 min. AMPK was maximally activated with a 3.5±1.1-fold (±s.e.m.) increase after 5 min of applied shear stress. AMPK activation was not increased in cells pre-incubated with the VE-cadherin function-blocking antibody (Fig. 1A,B).

As a second measure, we suppressed VE-cadherin expression by using RNA interference. To this end, lentiviruses encoding shRNAs against VE-cadherin (shVE-cadherin) or a scramble sequence (shScramble) as a control were generated and transduced into HUVECs. At 72 h after viral infection, VE-cadherin expression was decreased by 54±11% (mean±s.e.m.; Fig. 1C,D) when compared to the parental HUVECs (wild type). To determine whether AMPK was activated when VE-cadherin levels were reduced, the cells were incubated with and without 12 dyne/cm^2^ of shear stress. AMPK phosphorylation was significantly reduced in the cells depleted of VE-cadherin (Fig. 1E,F), confirming the essential role of VE-cadherin in mechanical transduction of the adherens junction complex and in facilitating AMPK activation in response to shear stress.

### LKB1 is required for mechanical activation of AMPK and eNOS

**Fig. 2.**
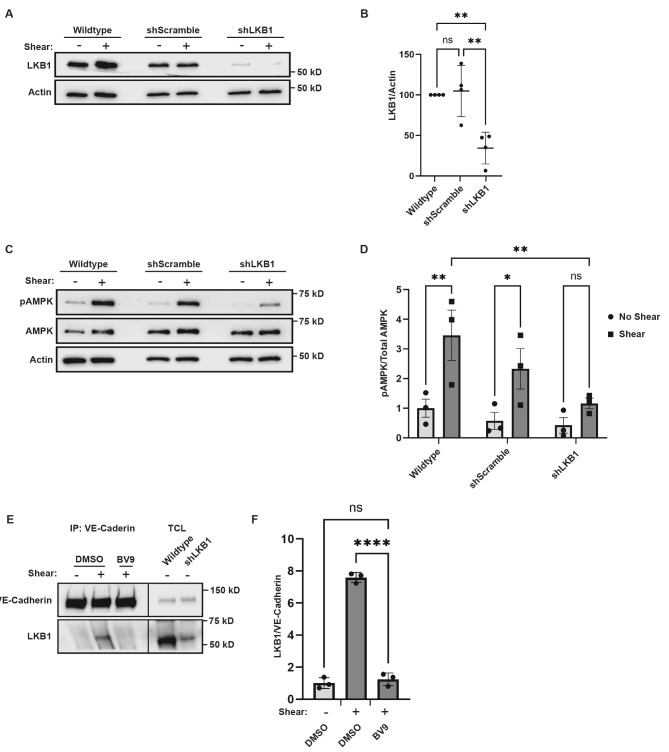
**LKB1 is the upstream kinase responsible for shear-induced AMPK activation.** (A,B) LKB1 inhibition using RNA interference. LKB1 expression was inhibited by treating HUVECs with lentiviruses encoding shRNAs targeting human LKB1 (shLKB1), or a scramble sequence (shScramble). Wild-type parental cells were employed as a control. Total cell lysates were immunoblotted with antibodies against LKB1 to show the level of inhibition or actin as a loading control. Representative western blots are shown in A. (B) The graph depicts the LKB1 levels normalized to the total protein levels; the data are mean±s.e.m., *n*=4 biologically independent samples. (C,D) Shear stress stimulated AMPK activation in cells depleted of LKB1. AMPK activity was monitored as described in the legend of Fig. 1A. In C, representative immunoblots are shown for cells under resting conditions (−) or exposed to 5 min of shear (+). The graph in D depicts the ratio of phosphorylated AMPK to total protein. The data are mean±s.e.m., *n*=3 biologically independent samples. (E,F) Shear-induced LKB1 coimmunoprecipitation with VE-cadherin. Cells were preincubated with DMSO or BV9 for 1 h and then were left resting (−) or exposed to shear stress (+) for 5 min. VE-cadherin was immunoprecipitated (IP), and the co-precipitation of LKB1 was examined by immunoblotting. The ratio of LKB1 recovered to VE-Cadherin immunoprecipitated is plotted in the graph (F). Data are mean±s.e.m., *n*=4 biologically independent samples. TCL, total cell lysates (5%). *P<0.05; **P<0.01; ****P<0.0001; ns, not significant [one-way ANOVA with Dunnett's test (B,F); two-way ANOVA with Tukey's multiple comparison test (D)].

AMPK can be targeted by three known kinases – LKB1, CaMKKβ (also known as CAMKK2) and TAK1 (also known as MAP3K7). Previous work implicated CaMKKβ in AMPK activation (Hurley et al., 2005). However, inhibiting CaMKKβ with the CaMKKβ inhibitor STO-609 did not prevent shear stress-induced AMPK activation (Fig. S2A,B). This led us to explore whether LKB1 might be responsible for AMPK activation under these conditions. To test the possibility that LKB1 facilitates AMPK activation in response to shear stress, we generated lentiviruses encoding shRNAs against LKB1 and infected HUVECs with the viral particles. Lentiviral application produced a 65±15% (mean±s.e.m.) decrease in LKB1 levels (Fig. 2A,B). Furthermore, this level of inhibition significantly decreased shear stress-induced AMPK activation (Fig. 2C,D).

Our observation that LKB1 is required for shear stress-induced AMPK and the observation that LKB1 is localized to the plasma membrane in response to shear stress (Bays et al., 2017; Dogliotti et al., 2017), suggested the possibility that LKB1 might be recruited to the cadherin adhesion complex. To test this possibility, LKB1 was recovered from cells that had been left resting or subjected to shear stress. VE-cadherin co-immunoprecipitation was examined by immunoblotting. Exposure to shear caused increased VE-cadherin binding to LKB1. Furthermore, the co-immunoprecipitation was lost upon pre-incubation of the cells with Bv9 function-blocking VE-cadherin antibody (Fig. 2E,F). These data suggest that LKB1 recruitment to VE-cadherin upon exposure to shear stress is required for AMPK activation.

**Fig. 3.**
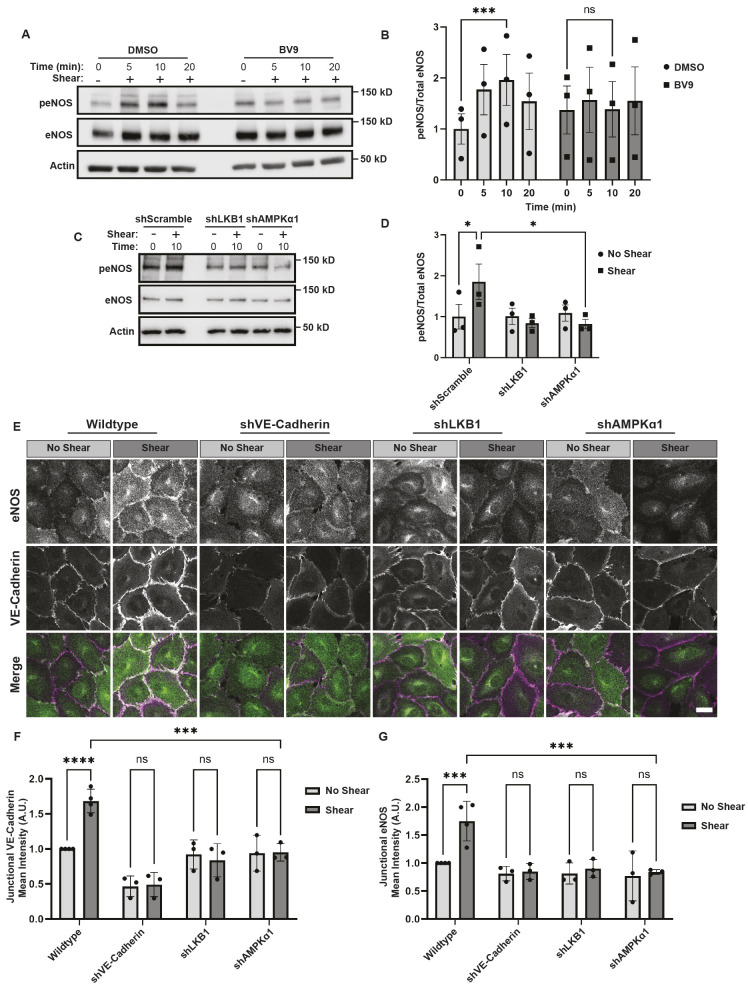
**VE-cadherin stimulated AMPK is required for eNOS membrane localization and activation.** (A–D) eNOS activation in the presence of (A,B) a VE-cadherin inhibitor or (C,D) shRNAs targeting signaling molecules downstream of VE-cadherin [i.e LKB1 (shLKB1), AMPKα1 (shAMPKα1) or a scramble sequence (shScramble)]. HUVECs were left untreated (DMSO) or treated with BV9 and left resting (−) or exposed to 12 dyne/cm^2^ orbital shear (+) for the indicated times in minutes. The cells were lysed and probed with an eNOS Ser1177 phospho-specific antibody (peNOS) or actin as a loading control. The blots were stripped and reprobed with antibodies that recognize total eNOS. Representative immunoblots are shown in A and C. The graphs in B and D depict the ratio of phosphorylated protein to total protein; the data are mean±s.e.m., *n*=3 biologically independent samples. (E–G) eNOS localization when VE-cadherin stimulated AMPK is disrupted. Wild-type HUVECs or those expressing shRNAs against VE-cadherin (shVE-Cadherin), LKB1 (shLKB1) or AMPKα1 (shAMPKα1) were left resting (no shear) or exposed to shear for 30 min. The cells were fixed and stained with antibodies against eNOS (green) or VE-cadherin (magenta) and examined by confocal microscopy. Representative images are shown in E. Scale bar: 20 μm. The graphs beneath the images represent the average corrected fluorescence intensity of VE-cadherin (F) or eNOS (G). The data are mean±s.d., *n*=3 biologically independent samples, of which two or three fields of view (FOV) were analyzed with >100 junctions per FOV. *P<0.05; ***P<0.001; ****P<0.0001; ns, not significant (two-way ANOVA with Tukey's multiple comparison test).

AMPK phosphorylates many downstream targets (Mihaylova and Shaw, 2011). One target of AMPK is eNOS (Morrow et al., 2003; Thors et al., 2011). When activated, eNOS generates the potent vasodilator nitric oxide, a molecule that is crucial for proper endothelial function (Durand and Gutterman, 2013). Thus, we next determined whether VE-cadherin and AMPK were required for eNOS activation. To address this possibility, cells were preincubated with the VE-cadherin function-blocking antibody BV9 (or a DMSO control) and left at rest or subjected to 12 dynes/cm^2^ shear stress. eNOS phosphorylation was assessed by blotting lysates with a phospho-specific antibody that targets Ser1177, a known AMPK phosphorylation site (Thors et al., 2011). After 10 min of shear stress, phosphorylation of Ser1177 was increased by 2.0±0.5-fold (mean±s.e.m.) in the DMSO-treated cells (Fig. 3A,B). By contrast, preincubation of cells with BV9 prevented a significant increase in eNOS phosphorylation (Fig. 3A,B). Given that LKB1 is required for VE-cadherin stimulation of AMPK, we explored whether LKB1 was required for eNOS activation triggered by shear stress. Inhibition of LKB1 using shRNAs decreased eNOS activation in response to shear stress (Fig. 3C,D).

To further assess the role of VE-cadherin and LKB1 in the mechanical activation of eNOS, we examined eNOS localization. eNOS myristylation and recruitment to the plasma membrane regulates its activity (Sakoda et al., 1995; Shaul et al., 1996), but the mechanism of this recruitment is not well defined. Given that VE-cadherin is highly expressed in the plasma membrane of endothelial cells, and stimulates AMPK to phosphorylate eNOS, we explored how VE-cadherin influences eNOS localization to adherens junctions. HUVECs were left resting or subjected to 12 dyne/cm^2^ of shear stress, and eNOS enrichment at cell–cell junctions was examined using confocal microscopy. eNOS was enriched in VE-cadherin cell–cell junctions in response to shear stress with a Pearson's correlation coefficient of 0.54±0.1 (mean±s.e.m.) (Fig. 3E–G; Fig. S3). This localization was dependent upon VE-cadherin, LKB1 and AMPKα1 (also known as PRKAA1), as their depletion reduced eNOS localization (Fig. 3E–G). Taken together, these findings indicate eNOS localization to cell–cell junctions requires not only VE-cadherin, but also LKB1-mediated activation of AMPK. These data reveal that mechanical cues transduced by VE-cadherin are required for eNOS activation and function.

### VE-cadherin-mediated AMPK activation is required for actin cytoskeletal reinforcement

**Fig. 4.**
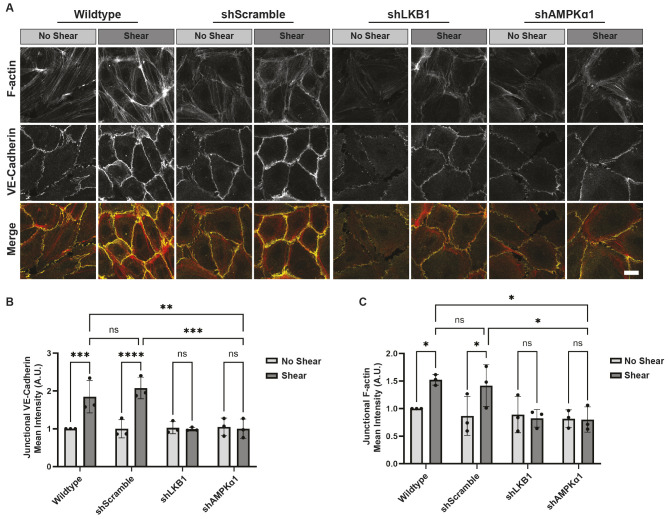
**LKB1 and AMPKα1 are required for shear-induced junctional reinforcement.** (A–C) Effect of AMPK inhibition of actin cytoskeletal reinforcement. Wild-type HUVECs or those expressing shRNAs against LKB1 (shLKB1), AMPKα1 (shAMPKα1) or a scrambled sequence (shScramble) were left resting (no shear) or exposed to shear for 30 min. The cells were fixed and stained with an antibody against VE-cadherin or phalloidin conjugated to Alexa Fluor 594 to visualize F-actin. Cells were examined by confocal microscopy. Representative images are shown in A. Scale bar: 20 μm. The graphs beneath the images represent the mean corrected fluorescence intensity of actin (B) or VE-cadherin (C) at the cellular junctions. The data are mean±s.d., *n*=3 biologically independent samples, of which two or three fields of view (FOV) were analyzed with >100 junctions per FOV. *P<0.05; **P<0.01; ***P<0.001; ****P<0.0001; ns, not significant (two-way ANOVA with Tukey's multiple comparison test).

Shear stress stimulates the reinforcement of the actin cytoskeleton and cell–cell adhesions. The requirement for AMPK in these events is unknown. To test a role for AMPK in junctional actin reinforcement, shear stress (12 dynes/cm^2^) or no shear stress was applied to confluent cultures of parental HUVECs (wild type) or HUVECs expressing shRNAs against LKB1 (shLKB1), AMPK (shAMPKα1) or a scramble sequence (shScramble) as a control. Junctional morphology and enrichment of the actin cytoskeleton were monitored via immunofluorescence. Under no shear stress conditions, both wild-type and shScramble cells displayed a fenestrated pattern of VE-cadherin staining, which shifted to a linear orientation upon application of shear stress (Fig. 4A). Additionally, VE-cadherin was enriched in junctions in response to shear stress. This is consistent with previous reports on the reinforcement of cell–cell junctions (Fig. 4A,B). The actin cytoskeleton was also reinforced when shear was applied (Fig. 4A,C). However, these structural reinforcements were lost when either LKB1 or AMPKα1 was depleted (Fig. 4A–C). Thus, VE-cadherin-mediated changes in LKB1 and AMPKα1 are required for reinforcement of the actin cytoskeletal and adherens junctions.

### VE-cadherin-mediated AMPK calls for increased metabolism

**Fig. 5.**
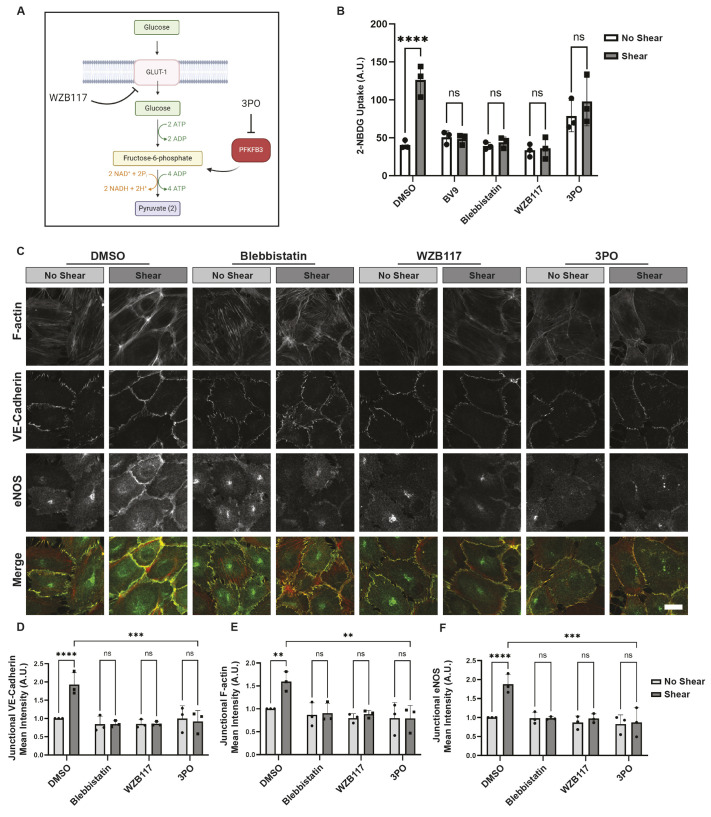
**Increased metabolism fuels endothelial reinforcement and eNOS localization to cell–cell junctions.** (A) Illustration of glycolytic inhibitors and their targets. (B) Mechanical or metabolic disruption and its effects on shear stress-stimulated glucose uptake. HUVECs were incubated in glucose free medium and were pretreated with DMSO or inhibitors against VE-cadherin (BV9), myosin II (blebbistatin), GLUT1 (WZB117) or phosphofructokinase fructose-bisphosphate 3 (3PO). The non-hydrolyzable glucose analog 2-NBDG was added to cells, and the cells were left resting or shear stress was applied for 1 h. Fluorometric analyses of the cell lysates were performed, and the amount of 2-NBDG uptake was quantified as the mean±s.e.m., *n*=3 biologically independent samples. (C) The effect of metabolic inhibitors on reinforcement of the actin cytoskeleton and enrichment of VE-cadherin and eNOS. HUVECs pretreated with DMSO or inhibitors against myosin II (blebbistatin), GLUT1 (WZB117) or phosphofructokinase fructose-bisphosphate 3 (3PO). Cells were then left under static (no shear) or exposed to shear stress (shear) conditions for 30 min. Cells were fixed and stained with Alexa Fluor 594–phalloidin (red) and antibodies against VE-cadherin (yellow) and eNOS (green). Representative images are shown in C. Scale bar: 20 μm. The graphs represent the average corrected fluorescence intensity of VE-cadherin (D), actin (E) and eNOS (F) in 100 junctions per condition. The data are mean±s.d., *n*=3 biologically independent samples, of which two or three fields of view (FOV) were analyzed with >100 junctions per FOV. **P<0.01; ***P<0.001; ****P<0.0001; ns, not significant [two-way ANOVA with Tukey's multiple comparison test (B); two-way ANOVA with Dunnett's multiple comparison test (D–F)].

Our observations that shear-induced stimulation of the adherens junctional complex increases AMPK activity prompts investigation into the consequences of this activation. The cellular response to mechanical cues requires elevations in enzymatic activity, actin polymerization and actomyosin contractility, all of which are energy-intensive processes necessary for cytoskeletal rearrangement and adhesion growth (Dawson et al., 2023). Given that glucose is the preferred energy source for endothelial cells, we hypothesized that the increase in AMPK activity due to VE-cadherin could enhance glucose metabolism. To begin to test this, glucose uptake was examined in HUVECs subjected to shear stress. For this, shear stress was applied to HUVECs, and the uptake of a fluorescently labeled, non-hydrolyzable 2-deoxyglucose (2-NBDG) analog was monitored. This analysis revealed that glucose uptake was increased 3.11-fold in response to the application of shear stress (Fig. 5A,B). This uptake specifically involved the most predominant glucose transporter in epithelia, GLUT1, as pre-treatment with WZB117, an inhibitor of GLUT1, blocked this effect (Fig. 5B). In contrast, preincubation of cells with 3PO, a phosphofructokinase-2 inhibitor that interferes with glucose metabolism had no effect on shear-induced glucose uptake. To determine whether this elevation in glucose uptake required VE-cadherin force transduction, cells were preincubated with a VE-cadherin function-blocking antibody or blebbistatin, a myosin II inhibitor. Both prevented glucose uptake, further suggesting that force transmission is required for elevated glucose uptake. These analyses reveal that the adherens junction complex is required for the elevated glucose uptake that was observed in response to the application of shear stress (Fig. 5B).

### VE-cadherin-mediated AMPK fuels cellular reinforcement and alignment

Having established that the adherens junctional complex stimulates glucose uptake, we next explored why glucose is crucial for cellular processes. Previous work indicates that elevations in glucose metabolism are required for a whole array of cellular responses (Pi et al., 2018). To test the possibility that glucose was required for increased reinforcement of cellular adhesions and the cytoskeleton, cells were treated with either WZB117, a small-molecule inhibitor of the glucose transporter GLUT1, or 3PO, a small-molecule inhibitor of PFKFB3, which inhibits glycolytic flux. The cells were then left under no shear or shear conditions and examined by confocal microscopy. A loss of junctional linearization, enrichment of VE-cadherin and actin cytoskeletal reinforcement was observed when GLUT1 or glycolysis was inhibited (Fig. 5C–E). Interestingly, another consequence of preventing glucose uptake or glycolysis was a loss of eNOS localization to cell–cell junctions (Fig. 5C,F). Thus, elevations in glucose uptake and metabolism are required for fortification of the cytoskeleton and adhesion of cells.

**Fig. 6.**
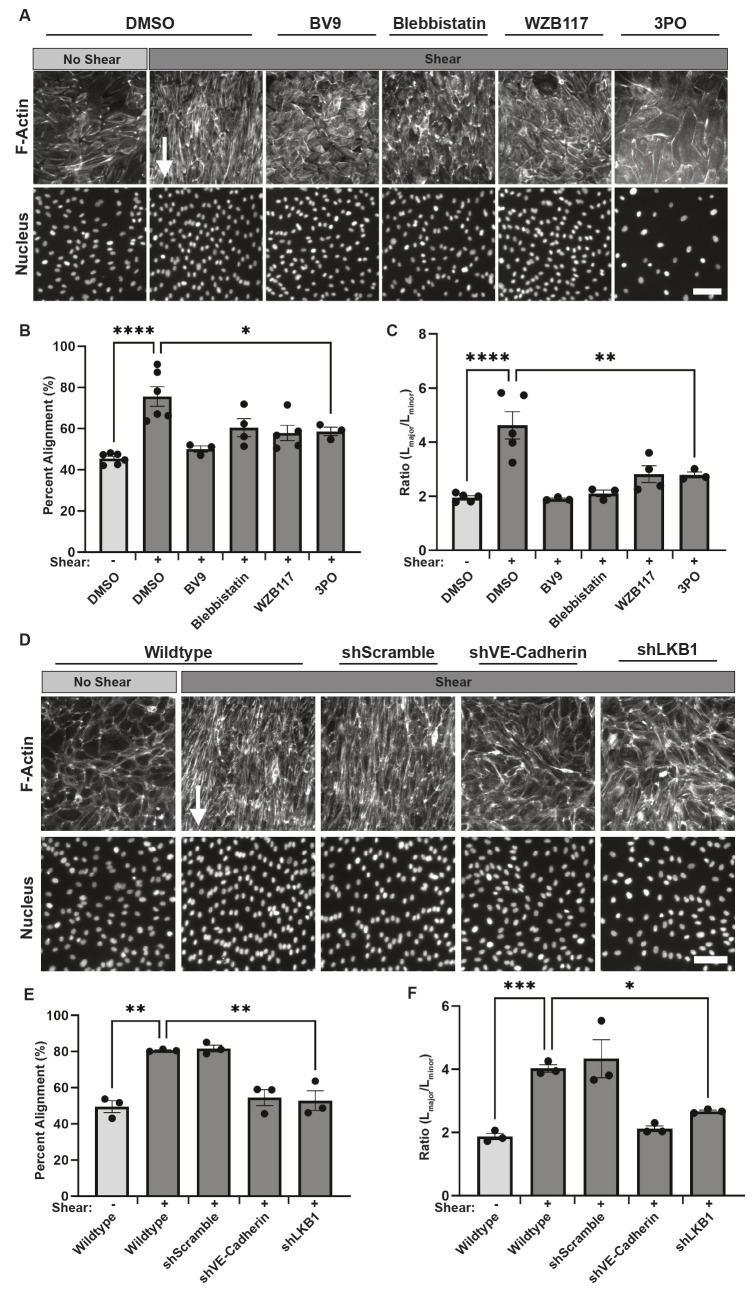
**Increased VE-cadherin mediated signaling to AMPK and metabolism support endothelial alignment.** The effects of metabolic inhibitors (A–C) or shRNAs against components of the signal transduction cascade initiated by VE-cadherin (D–F) on endothelial alignment. HUVECs pretreated with DMSO or inhibitors against VE-cadherin (BV9), myosin II (blebbistatin), GLUT1 (WZB117) or phosphofructokinase fructose-bisphosphate 3 (3PO) (A–C) or HUVECs expressing shRNAs against VE-cadherin (shVE-cadherin), LKB1 (shLKB1) or a scramble sequence (shScramble) were then left resting (no shear) or exposed to shear stress (shear) for 48 h. For cells treated with inhibitors, new medium containing a fresh supply of inhibitors was added after 24 h. Cells were fixed and stained with Alexa Fluor 594–phalloidin and DAPI to visualize actin and the nucleus, respectively. Representative images are shown in A and D. Scale bars: 40 μm. Alignment was assessed based on whether the major axis of the cell and its Feret angle was within ±45° of the direction of flow and is quantified in B and E. Elongation was assessed by comparing the ratio of the length of the major axis (L_major_) with the width of the minor axis (L_minor_) and is quantified in C and F. >250 cells across three fields of view (FOV) per condition were assessed for alignment and 100 cells across three FOV per condition were assessed for elongation. The average percentage alignment and elongation is plotted as the mean ±s.e.m., *n*=3–5 biologically independent experiments. *P<0.05; **P<0.01; ***P<0.001; ****P<0.0001 (one-way ANOVA with Dunnett's multiple comparison test).

A common *in vitro* and *in vivo* physiological response of endothelial cells is for them to align parallel to the direction of shear stress, an event that is crucial in the protection against the development of atherosclerosis and inflammation (Immanuel and Yun, 2023). To test whether the adherens junction complex-activated metabolism we studied herein is important for endothelial cell alignment, HUVECs were left resting or subjected to shear stress for 48 h. The cells were stained to visualize actin stress fiber alignment and nuclei orientation. Alignment was assessed by measuring the Feret angle of the major axis (L_major_) of the cells with respect to the direction of the flow. We found that 77.5±5.1% (mean±s.e.m.) of DMSO-treated cells exposed to shear were aligned. However, disruption of mechanotransduction by non-muscle myosin II inhibition with blebbistatin or VE-cadherin disruption using BV9 significantly reduced cellular alignment (Fig. 6A,B). Inhibition of glucose uptake or glycolysis also decreased alignment (Fig. 6A,B; Fig. S4A). We sought to determine whether the loss of alignment was a consequence of decreased elongation by measuring the ratio between the major (L_major_) and minor (L_minor_) axis of the cells. All inhibitors prevented cellular elongation (Fig. 6C). As a second measure, we disrupted VE-cadherin (shVE-cadherin) or LKB1 (shLKB1) and assessed alignment using wild-type cells or cells expressing a non-targeting RNA (shScramble) as a control (Fig. 6D). Loss of VE-cadherin or LKB1 significantly reduced alignment and elongation as compared to the wild-type and shScramble controls (Fig. 6E,F). Collectively, these findings suggest that shear stress activation of the adherens junctional complex induces LKB1, which then activates AMPK. This activation of AMPK leads to increased glucose uptake, fueling the structural reinforcement necessary for cells to align in response to mechanical forces.

## DISCUSSION

The response of cells to mechanical cues requires increases in protein synthesis, transport, actomyosin contractility and actin polymerization, which are needed for the cell to counter the applied tension (Dawson et al., 2023). All these processes require energy. In epithelia, increased tension is propagated across E-cadherin and signals for increased AMPK activity, which stimulates a downstream signal transduction cascade that culminates in increased contractility (Bays et al., 2017). Here, we report the existence of a similar pathway in endothelia. Indeed, shear stress stimulated the activation of AMPK. Although endothelial cells express a mechanosensitive complex that includes the transmembrane adhesion protein PECAM-1, VEGFR and VE-cadherin, the loss of VE-cadherin disrupts this complex. Here, we show that AMPK activation is dependent on a functioning adherens junction complex, which stimulates eNOS activity. These findings not only confirm the linkage between cell mechanics and metabolism, as observed in epithelial cells but also extend this relationship to include eNOS activity and vasodilation, further expanding the emerging paradigm of how mechanical and metabolic processes are interconnected.

What is the increase in metabolism needed for? Numerous independent studies have demonstrated that the actin cytoskeleton is a major energetic demand in cells (Daniel et al., 1986; Bernstein and Bamburg, 2003; Gómez-Escudero et al., 2019) and that altered cytoskeletal dynamics affect cell metabolism (Dawson et al., 2023). We previously reported that increased metabolism in epithelial cells supports cytoskeletal rearrangements and growth of the adhesion complex in response to tension (Salvi et al., 2021). Here, we find that perturbing force-stimulated metabolism blocks actin cytoskeletal reinforcement, reinforcement of the cadherin adhesion complex, and cellular alignment (Figs 4–6). All these events require robust actin cytoskeletal changes and are blocked by inhibitors of cellular metabolism, thereby suggesting a requirement for increased metabolism to support the actin cytoskeleton.

The notion that energy is required to support the actin cytoskeleton has recently been challenged (Holland and Gallo, 2023). That study employed a genetically encoded biosensor that indicates the ATP level based on the ratio of ADP to ATP and reported that ATP/ADP ratios remained relatively constant when growth cones were stimulated with nerve growth factor, which increased actin dynamics. The authors also presented evidence that inhibiting actin cytoskeletal rearrangements had no effect on ATP/ADP ratios. Although the biosensors used in the Holland and Gallo (2023) study have been useful in some settings, they report on ADP/ATP ratios, not ATP concentrations. Thus, they are susceptible to optical overlap with cellular sources of auto-fluorescence, which is expected to be high in a growth cone (Lobas et al., 2019). Nonetheless, these studies raise the possibility that the energy demands for reinforcing the cytoskeleton and adhesion complex, as well as for cellular alignment, might be met by other energy-intensive processes, such as protein phosphorylation, transport and synthesis. Further research is needed to clarify which of these processes are essential when a cell is under tension.

Unlike many other cell types, endothelial cells can increase vasodilation through the synthesis and release of vasoactive substances, such as nitric oxide, prostacyclin and endothelium-derived hyperpolarizing factor (Durand and Gutterman, 2013). Here, we show a 2-fold increase in eNOS phosphorylation in cells under shear stress (Fig. 3; Fig. S1), corroborating previous findings that shear stress stimulates eNOS activity (Nishida et al., 1992; Kolluru et al., 2010; Thors et al., 2011). Importantly, our results show that this increased eNOS activation requires VE-cadherin (Fig. 3), a novel finding not previously reported. Prior to this study, shear-stimulated increases in eNOS activity were known to occur in response to increased ion channel conductance (Ungvari et al., 2001), VEGF treatment (Feliers et al., 2005) and thrombin activation (Thors et al., 2008). The involvement of VE-cadherin we report here adds a significant new dimension to the understanding of eNOS regulation under shear stress.

In addition to requiring VE-cadherin to maintain the adherens junction complex, shear stress-stimulated increases in eNOS phosphorylation required AMPK (Fig. 3), and other work has demonstrated that eNOS is a direct substrate for AMPK (Thors et al., 2011). Collectively, these data suggest the existence of a mechanosignaling pathway whereby VE-cadherin-stimulated AMPK phosphorylates eNOS to increase NO production and elevate vasodilation. In this scenario, mechanics, metabolism and vasodilation are linked. The importance of coupling mechanical and metabolic signaling with vasodilation remains speculative. Given that vasodilation enhances the flow of blood to areas of the body that lack nutrients, it is tempting to speculate that mechanical signaling is linked to vasodilation to elevate the delivery of these nutrients.

Emerging evidence suggests that the links between mechanics, metabolism and vasodilation are much more complex than presented herein. An RNAi screen aimed at identifying cadherin interacting proteins unveiled 400 proteins organized into 17 regulatory hubs, with the metabolism hub being the third largest (Toret et al., 2014). Additionally, a complex relationship between Rho kinase, a key mediator of contractility, and the metabolic regulator, AMPK, has emerged (Noda et al., 2014; Huang et al., 2018). Finally, our current studies revealed that inhibition of glycolysis decreases eNOS localization to the plasma membrane, implying that without the generation of glycolytic intermediates, there is less need for increased vasodilation to transport nutrients. This is likely not the only mechanism for coordinating the activities of these interlinked pathways as eNOS interacts and S-nitrosylates pyruvate kinase M2, which ultimately decreases substrate flux through the pentose phosphate pathway, thereby decreasing the production of NADPH, an important reducing equivalent (Siragusa et al., 2019). More work is needed to unravel the complexities of the linkages between mechanics, metabolism and vasodilation to better understand their regulation.

## MATERIALS AND METHODS

### Cell lines

Human umbilical vein endothelial cells (HUVECs, Lonza Scientific) and bovine aortic endothelial cells (BAECs, American Tissue Culture Collection) were maintained in MCDB 131 medium (Sigma-Aldrich), supplemented with 5% heat-inactivated FBS (R&D Systems), 12 µg/ml bovine brain extract (Lonza CC-4098), 10 ng/ml hEGF (Sigma E9644), 1 µg/ml hydrocortisone (Thermo Fisher Scientific), 200 µg/ml ascorbic acid (Sigma), and 5% penicillin-streptomycin (GIBRO-BRL). All cell lines were used for no more than 6 passages and periodically checked for mycoplasma contamination. The 293FT cells (Invitrogen) are virus-producing cells that are a derivative of 293T cells and were maintained in DMEM supplemented with 10% heat-inactivated FBS, 0.1 mM non-essential amino acids (GIBCO-BRL), 6 mM L-glutamine, 1 mM MEM sodium pyruvate, and 1% penicillin-streptomycin and 500 µg/ml geneticin (GIBCO-BRL).

### Constructs

To inhibit the expression of proteins using small hairpin RNAs, the following cDNA sequences were cloned into pLKO.1 (Addgene plasmid #10878, deposited by David Root) by Genscript Corporation: shVE-cadherin, 5′-CCGGAGATGCAGAGGCTCATGATCTCGAGATCATGAGCCTCTGCATCTTTTTTG-3′; shLKB1, 5′-CCGGCGAAGAGAAGCAGAAAATGCTCGAGCATTTTCTGCTTCTCTTCGTTTTTG-3′; shAMPKα1, 5′-CCGGGAGGAGAGCTATTTGATTACTCGAGTAATCAAATAGCTCTCCTCTTTTTG-3′; and shScramble, 5′ CCGGCCTAAGGTTAAGTCGCCCTCGCTCGAGCGAGGGCGACTTAACCTTAGGTTTTTG-3′.

### Viral production and transduction

Lentiviral particles were produced in 293FT cells according to the manufacturer's instructions. HUVECs were grown to ∼50% confluency. On the day of infection, cells were washed twice with PBS and incubated with viral supernatant containing 7 µg/ml of polybrene for 15–18 h. After incubation, the cultures were washed, and full growth medium was added. The infected cells were employed 48–72 h after infection.

### Orbital shear stress

Orbital shear stress was applied to 90–95% confluent cells in growth medium at the indicated amplitudes and durations by incubating samples on an orbital shaker at 37°C and 5% CO_2_.

### Inhibitors and function-blocking antibodies

Inhibitors were incubated with the cells 1 h prior to application of orbital shear stress. CAMKKβ was inhibited using 10 µg/ml STO-609 (Selleck Chemicals). Myosin II was inhibited using 50 μM blebbistatin (Sigma). VE-cadherin was inhibited using 4 µg/ml BV9 (Invitrogen). GLUT-1 was inhibited using 30 nM WZB117 (Tocris Biosciences). PFKFBP was inhibited using 25 µM 3PO (Millipore).

### Immunoprecipitation and immunoblotting

For immunoprecipitation of VE-cadherin, cells were lysed in 1× RIPA (50 mM Tris-HCl pH 7.6, 150 mM NaCl, 1% NP-40, 0.5% Sodium Deoxycholate, 0.1% SDS, 50 mM NaF, 2 mM EDTA, 20 μg/ml aprotinin, 2 mM sodium vanadate and 1 mM PMSF). The lysates were clarified at 12,000 ***g*** for 15 min at 4°C. The supernatant was collected, and 2 µg of VE-cadherin (14-1449-82, Invitrogen) antibody was added and incubated for 2 h at 4°C with rotation. The complexes were recovered using protein A beads, washed three times in lysis buffer, and resuspended in 2× sample buffer (200 mM Tris-HCl pH 6.8, 20% glycerol, 5% β-mercaptoethanol, 4% SDS and 0.3% Bromophenol Blue). Samples were boiled for 10 min and resolved using SDS-PAGE and transferred to PVDF (Immobilon). The membranes were blocked in 5% BSA for phosphor-AMPK, AMPK, pENOS, eNOS, VE-cadherin or 5% non-fat milk (Hy-Vee) for β-catenin, β-actin and GAPDH. The membranes were incubated with the primary antibodies overnight at 4°C. Primary antibodies used for immunoblotting were: monoclonal Thr-172 phospho-AMPK at 1:1000 (Cell Signaling; 40H9), monoclonal AMPK-α at 1:1000 (Cell Signaling; 2532), monoclonal Ser-1177 phospho-eNOS at 1:500 (Cell Signaling; 9571S), monoclonal eNOS at 1:500 (Cell Signaling; D9A5L), monoclonal VE-cadherin at 1:1000 (Cell Signaling; D87F2), monoclonal β-actin at 1:1000 (Cell Signaling; 8H10D10), monoclonal β-catenin at 1:1000 (BD Transduction; 610153) and monoclonal LKB1 at 1:1000 (Cell Signaling; 27D10; or Abcam EPR19379 for coimmunoprecipitation studies). The membranes were washed three times in TBST and blocked in either 5% BSA or 5% milk and incubated with anti-mouse-IgG conjugated to HRP (Jackson Labs) or anti-rabbit HRP (Jackson Labs) at a 1:1000 dilution for 1 h at room temperature. The membranes were washed three times in TBST and visualized by using chemiluminescence detection reagent (Pierce), and signal was detected on an Odyssey Fc Imager (Li-Cor Biosciences). For analysis, the integrated density of each band was measured using the Image Studio Lite Ver 5.2 software. Ratios of the phosphorylated compared to the total proteins were quantified by stripping and re-probing membranes. Quantification of each assay represents a minimum of three experiments. Quantification was undertaken using Studio Lite v. 5.2 software and statistical analysis was conducted using GraphPad Prism 10.1.0 using a two-tailed unpaired Student's *t*-test when comparing two conditions, a one-way ANOVA with Dunnett's comparison analysis when comparing more than two conditions, or a two-way ANOVA with Tukey's analysis when comparing assays with multiple conditions and two variables. Uncropped images of western blot from this paper are shown in Figs S5 and S6.

### Immunofluorescence

Coverslips were coated with human fibronectin at 10 μg/ml, and cells were plated and allowed to expand to confluency. Cells were fixed with 4% paraformaldehyde, permeabilized with 0.1% Triton X-100 and washed with PBS. Cells were blocked with 10% BSA (Sigma) in PBS. The following reagents and primary antibodies were diluted in 10% BSA (Research Products International) and allowed to incubate overnight: phalloidin conjugated with Alexa Fluor 594 was used to visualize actin, 1:1000, DAPI was used to visualize the nucleus, 1:1000; monoclonal anti-VE-cadherin antibody, 1:500 (Invitrogen; 14-1449-82); monoclonal anti-eNOS, 1:250 (Cell Signaling; D9A5L); and monoclonal anti-Pecam-1, 1:500 (Cell Signaling; 89C2). Cells were washed with PBS, and the anti-rabbit-IgG conjugated to Alexa Fluor 488 and/or anti-mouse-IgG conjugated to Alexa Fluor secondary antibodies were diluted 1:1000 in 10% BSA and allowed to incubate for 2 h at room temperature. Cells were washed with PBS and mounted on coverslips using MOWIAL (Sigma).

### Immunofluorescence acquisition and quantification

Fluorescence images were captured at room temperature with a confocal microscope (model LSM 710; Carl Zeiss Micro Imaging, Inc.). A 40× or 60× oil objective (Carl Zeiss Micro Imaging, Inc.) with a numerical aperture of 1.3 was used. Images were obtained using the Zen2009 software (Carl Zeiss Micro Imaging). Laser settings were kept identical for all conditions for each individual experiment. To assess junctions, a single image from the *Z*-plane was acquired, and the intensity of this plane was quantified. Quantifications of images were made using ImageJ. For quantification, the images were preprocessed by separating the channels and then subtracting the background using a rolling ball radius of 50 pixels. To determine the enrichment of proteins at the cellular junctions, <100 discrete randomly chosen junctions were measured across three fields of view. Individual junctions were measured by creating a region of interest (ROI) fit to the junction using VE-cadherin as a reference. Graphs report the distribution of the intensities from a single experiment across three fields of view. The experiments were repeated in triplicates, and similar results were obtained.

### Cellular alignment

Cells were grown to 70–80% confluency on surfaces that were coated with 10 μg/ml of purified human fibronectin (Sigma-Aldrich). Orbital shear stress was applied to the cells for 48 h at 37°C and 5% CO_2_. The cells were then washed twice with PBS and fixed in 4% paraformaldehyde. Cells were washed and incubated in PBS containing 10% BSA containing 1:1000 Alexa-Fluor-594-conjugated phalloidin and 1:1000 DAPI for 2–4 h at room temperature. Images were captured at room temperature with an inverted microscope (Axiovert 200 M; Carl Zeiss), equipped with an ORCA-ERA 1394 HD camera (Hamamatsu Photonics, Hamamatsu City, Japan). A 10× Plan Neofluor objective (NA 0.55; Carl Zeiss) was used to capture fluorescent images.

### Assessing endothelial alignment

ImageJ v1.54 was used to calculate cellular alignment. Briefly, masks were created from the nuclear outline determined from the DAPI image, by adaptive thresholding. The ‘Analyze Particles’ feature was then used to quantify the parameters of the binary image. Here, a 0.0005–0.01 particle size threshold was used to remove nuclei that were overlapping or smaller artifacts. The Feret's diameter was assessed, and the Feret angle was used to assess cellular orientation. A cell was determined to be aligned to the direction of flow if the Feret angle was ±45° centered on the known direction of flow. As a second measure of cellular alignment, actin fiber orientation was measured in the same manner. There were no significant differences between methods.

### Glucose uptake assay

Glucose uptake was assessed by measuring the fluorescence of 2-NBDG (Cayman Chemicals 11046). Cells were grown to confluency. Cells were pretreated with inhibitors or function-blocking antibodies for 1 h in glucose-free medium with 0.1 mg/ml of 2-NBDG. Cells were then left under no shear or shear conditions for an additional hour. After incubation time the cells were washed three times with PBS and lysed in EB lysis buffer (1 mM Tris-HCl, pH 7.6, 50 mM NaCl, 1% Triton X-100, 5 mM EDTA, 50 mM NaF, 20 μg/ml aprotinin, 2 mM Na_3_VO_4_ and 1 mM PMSF). Lysate was clarified by spinning down for 10 min at 12,000 ***g***. The supernatant was transferred to 96-well plates, and a fluorescence reading at 485 nm and 535 nm was taken, and the ratio of those determined (Biotek Synergy Neo model NEOALHPA B, Gen 5 software).

### Statistics and reproducibility

Statistical differences between groups of data were analyzed using a series of two-tailed unpaired Student's *t*-tests for assays that contained only two conditions, a one-way ANOVA with Dunnett's test when comparing more than two conditions, and a two-way ANOVA with Tukey's analysis when comparing assays with more multiple conditions and variables. All statistical analysis and data graphing was undertaken on Prism (Version 10.1.0). All quantified experiments represent at least three biologically independent samples.

## Supplementary Material



10.1242/joces.262232_sup1 Supplementary information
