# Supplementary material for: Shear stress-stimulated AMPK couples endothelial cell mechanics, metabolism and vasodilation
Source: J Cell Sci. 2024 Dec 18;137(24):jcs262232. doi: 10.1242/jcs.262232 (PMC11795286; doi:10.1242/jcs.262232)
Supplement: Supplementary information [file joces-137-262232-s1.pdf]

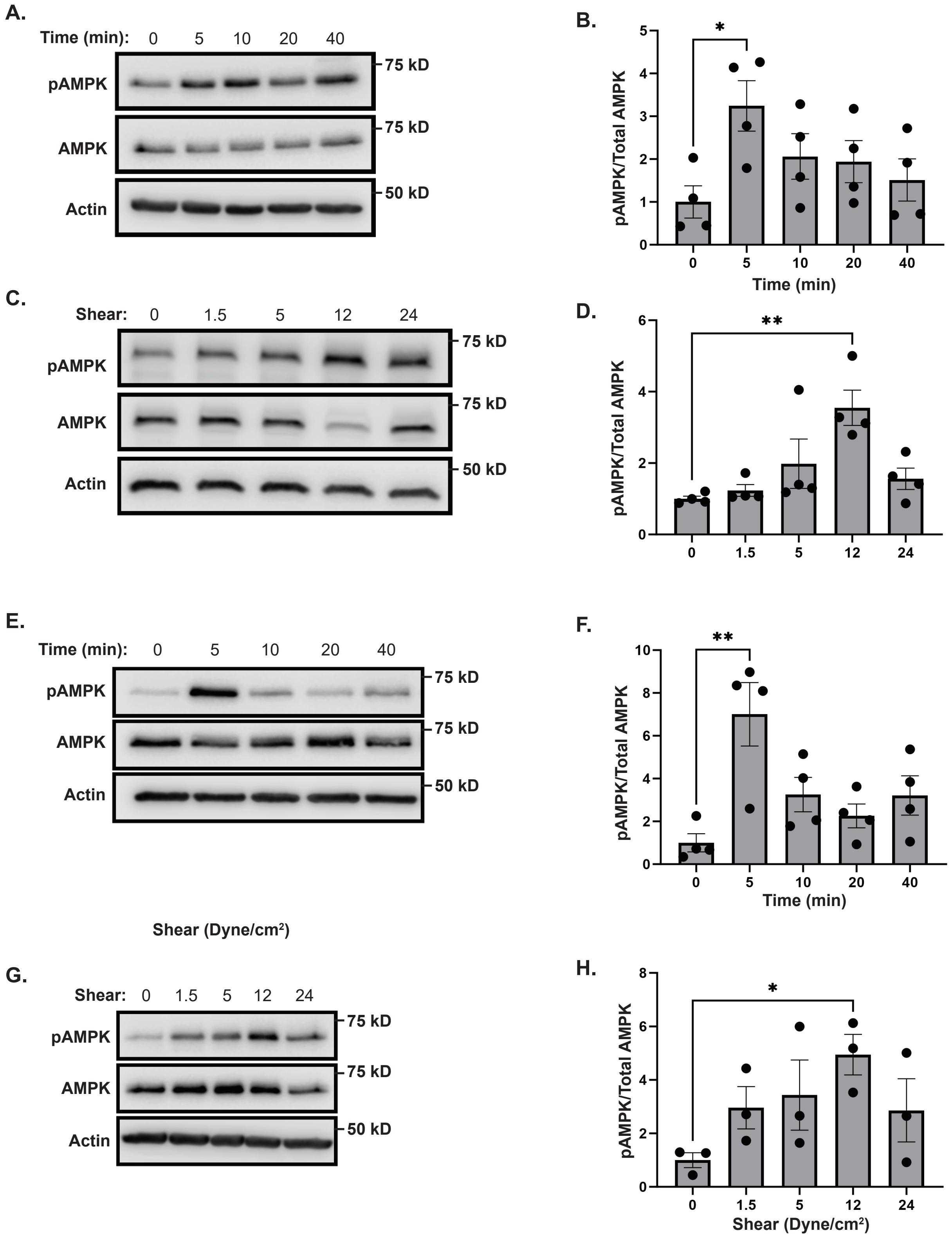

Fig. S1. Effects of shear stress on AMPK act vat on. The time dependent activation of AMPK. HUVECs (A-D) or BAECs (E-H) were left resting or exposed to shear stress for the indicated times (A-B,E-F) or the indicated levels of shear stress in dyne/cm2 (C-D,G-H). AMPK activation was examined by immunoblotting total cell lysates with an antibody that recognizes AMPK phosphorylation in its activation loop (pAMPK). Actin was blotted as a loading control. The blots were stripped and probed with antibodies that report on total AMPK levels (AMPK). Representative immunoblots are shown in A, C, E, and G. The graphs (B,D,F,H) depict the quantification of the ratios of phosphorylated AMPK to total AMPK; the data are mean ±s.e.m., n =3-4 biologically independent samples. *$p<0.05$, **$p<0.01$, (one-way ANOVA with a Dunnett's comparison test).

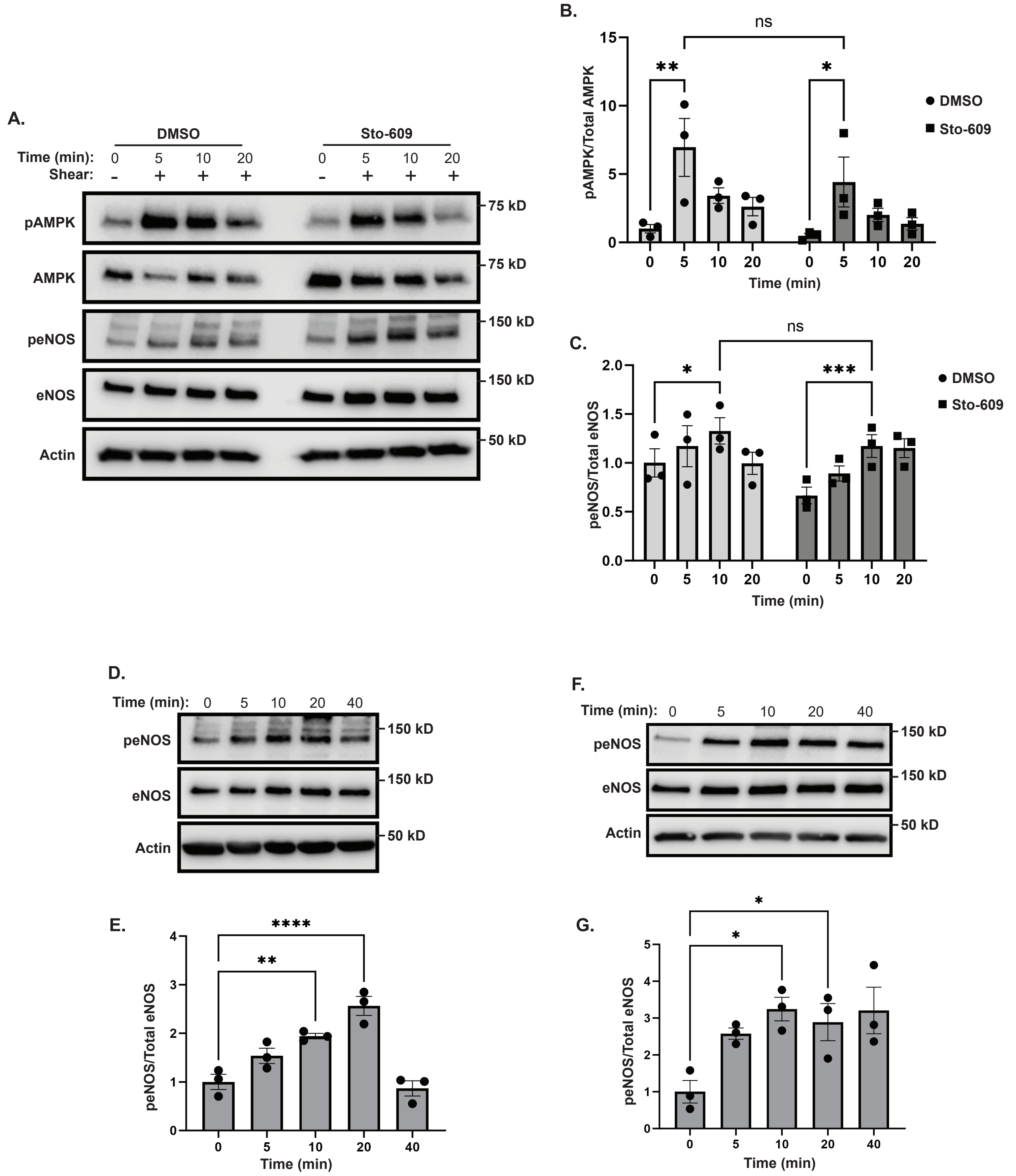
A.
DMSO
Sto-609
Time (min): 0 5 10 20 0 5 10 20
Shear: - + + + - + + +
pAMPK
AMPK
peNOS
eNOS
Actin
75 kD
75 kD
150 kD
150 kD
50 kD
B.
pAMPK/Total AMPK
ns
**
*
DMSO
Sto-609
Time (min)
C.
peNOS/Total eNOS
ns
*
***
DMSO
Sto-609
Time (min)
D.
Time (min): 0 5 10 20 40
peNOS
eNOS
Actin
150 kD
150 kD
50 kD
E.
peNOS/Total eNOS
****
**
Time (min)
F.
Time (min): 0 5 10 20 40
peNOS
eNOS
Actin
150 kD
150 kD
50 kD
G.
peNOS/Total eNOS
*
*
Time (min)

Fig. S2. Effects of shear stress on eNOS activation. (A-C). Shear stimulated AMPK and eNOS activation in the presence of the CaMKKβ inhibitor STO-609. HUVECs were left resting (-) or exposed to shear stress (+) for the indicated times in the presence or absence of STO-609 or DMSO as a control. AMPK and eNOS activation were examined by immunoblotting total cell lysates with an antibody that recognizes AMPK phosphorylation in its activation loop (pAMPK) or phosphorylated Ser1177 on (peNOS). Actin was blotted as a loading control. The blots were stripped and probed with antibodies that report on total AMPK (AMPK) or eNOS. The graph in B depicts the ratios of phosphorylated AMPK to total AMPK while the graph in C illustrates the ratios of phosphorylated eNOS to total eNOS; the data are mean ±s.e.m., n =3 biologically independent samples. *p<0.05, **p<0.01, ***p<0.001, (two-way ANOVA, with a Tukey's comparison test). (D-G) The effects of shear stress amplitude and time on eNOS activation. HUVECs were left resting or exposed to shear stress for the indicated times (D-E) or the indicated levels of shear stress in dyne/cm2 (F-G). eNOS activation was examined by immunoblotting total cell lysates with an antibody that recognizes eNOS phosphorylated on Ser1177 (peNOS). Actin was blotted as a loading control. The blots were stripped and probed with antibodies that report on total eNOS levels (eNOS). The graphs are the quantification of the ratios of phosphorylated eNOS to total eNOS respectively; the data are mean ±s.e.m., n =3 biologically independent samples. *p<0.05, **p<0.01, (one-way ANOVA with a Dunnett's comparison test), ns = not significant.

Journal of Cell Science • Supplementary information

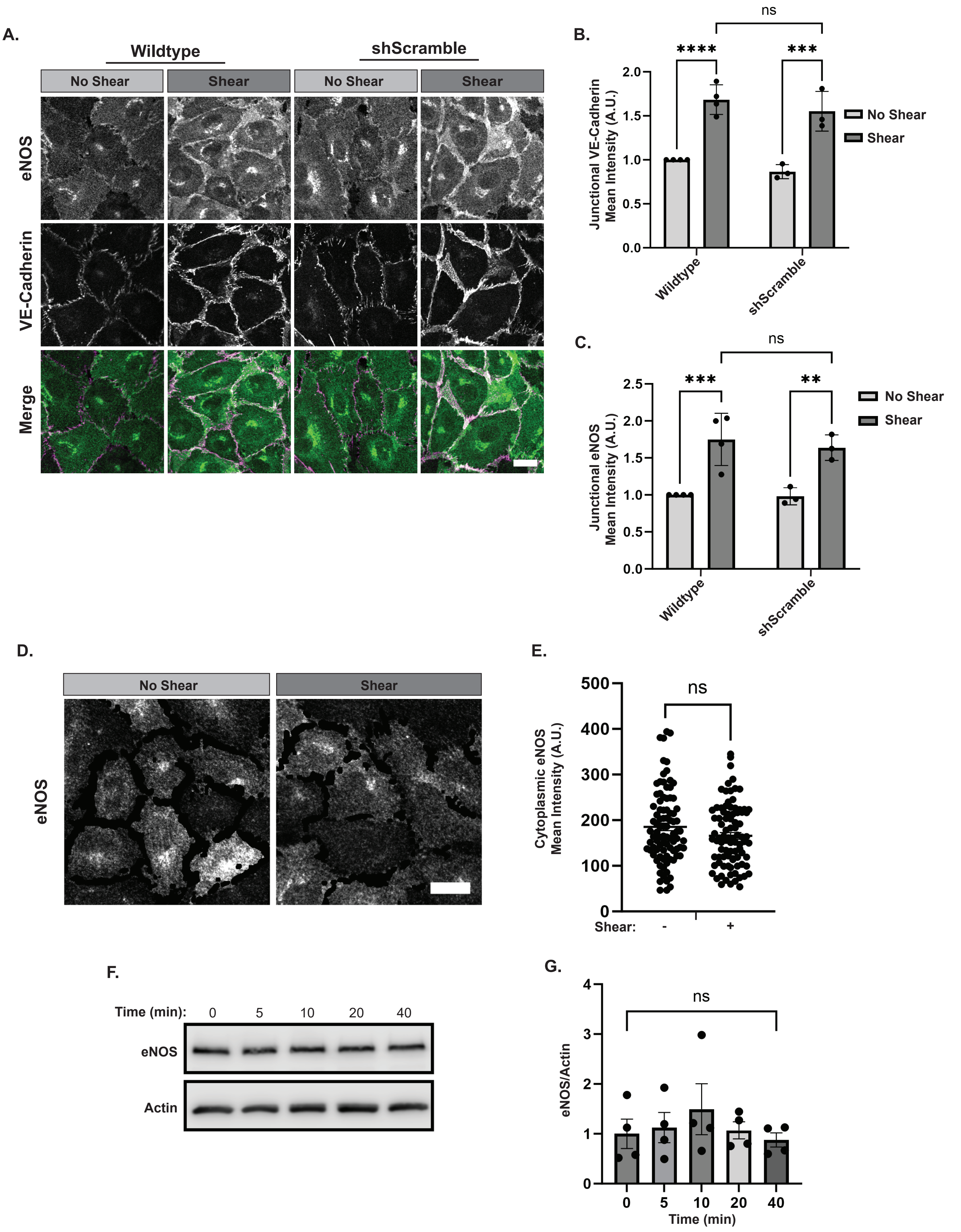

Journal of Cell Science • Supplementary information

Fig. S3. Effects of shear stress on eNOS localization and expression. (A-C) The scramble controls. HUVECs with and without shear stress applied were stained and analyzed as described in Figure 3E. Representative images are shown in A and the quantification of the junctional VE-cadherin is shown in B and eNOS C in 100 junctions per condition and cell type. The data are mean ± S.D., n=3 biologically independent samples, of which 2-3 FOV were analyzed with >100 junctions per FOV. **p<0.01, ***p<0.001 ****p<0.0001 (Two-way ANOVA, with Tukey's multiple comparison test). Scale bar = 20 um. (D and E) the relative abundance of cytoplasmic eNOS was measured by first creating a mask using VE-Cadherin fluorescence (not shown). This mask was then used to distinguish between junctional or cytoplasmic eNOS as shown in D. Scale bar = 20 µm. Figure (E) is the quantification of the cytoplasmic eNOS of 90 individual cells across three images. An unpaired t-test was utilized to determine if the populations were significantly different and found not to be (ns) = not significant. (F and G) Time dependent protein expression of eNOS. eNOS expression during the first 40 minutes of shear stress application was monitored by immunoblotting total cell lysates with antibodies against eNOS. Actin was blotted as a loading control. A representative immunoblot is shown in E and the graph in F is the quantification of total eNOS expression as a function of the loading control; the data are mean ±s.e.m., n =4 biologically independent samples. (ns) denotes not significant.

Journal of Cell Science • Supplementary information

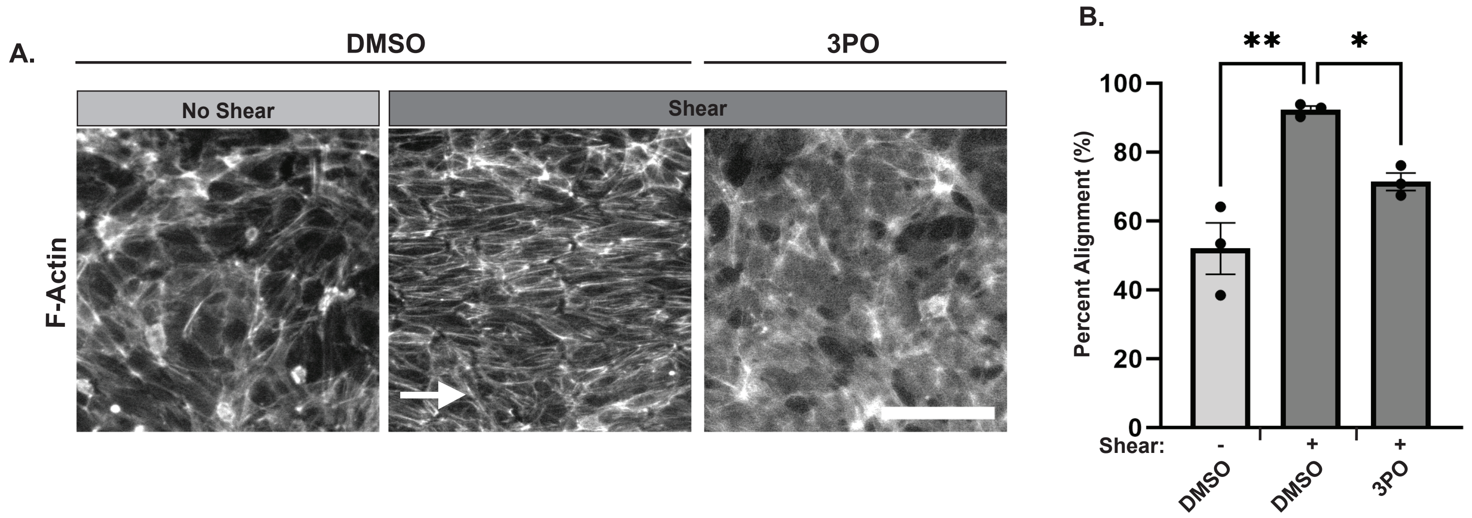

Fig. S4. Effects of 3PO on endothelial cell alignment. (A and B) Cells were seeded at a high density and then treated with DMSO as a control or 3PO. Cells were exposed to static (no shear) or shear conditions for 48 hours. Cells were fixed and stained with DAPI (nucleus) and phalloidin conjugated to Alexa594 (F-actin). Representative images are shown in A. Scale bar = 80 um. Alignment was quantified as detailed in the legend for Figure 6A, and the average percent alignment was plotted as the mean ±s.e.m., n=3 biologically independent experiments. **p<0.01, ***p<0.001 (One-way ANOVA, with Dunnett's multiple comparison test.

Journal of Cell Science • Supplementary information

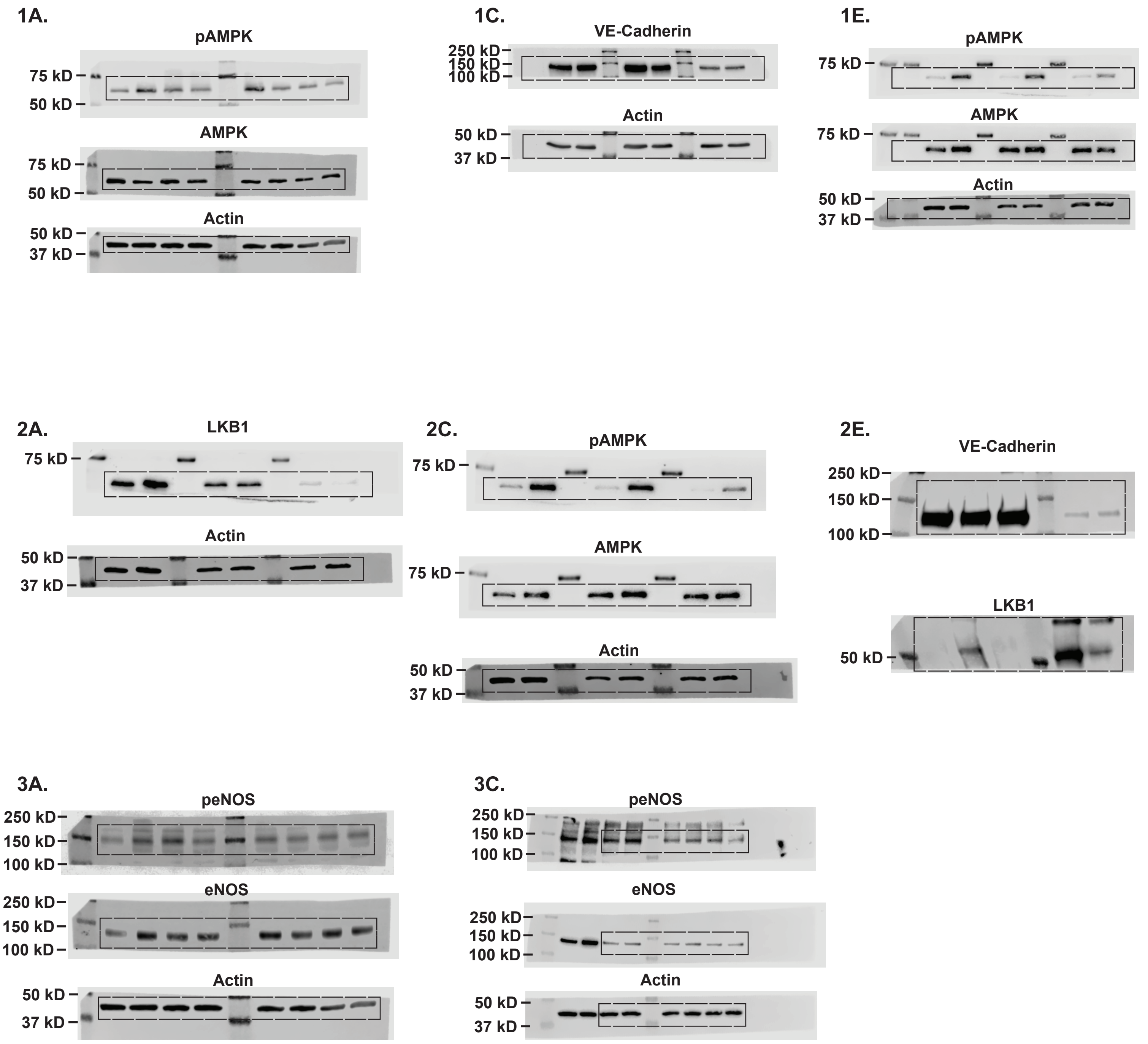

Fig. S5. Uncropped Blots for Figures 1, 2, and 3: The uncropped blots are shown, and include channels that identify the ladder. The dotted region indicates the portion of the blot included in the figure. The Actin blot used in 1A and 3A are identical as they are from the same gel.

Journal of Cell Science • Supplementary information

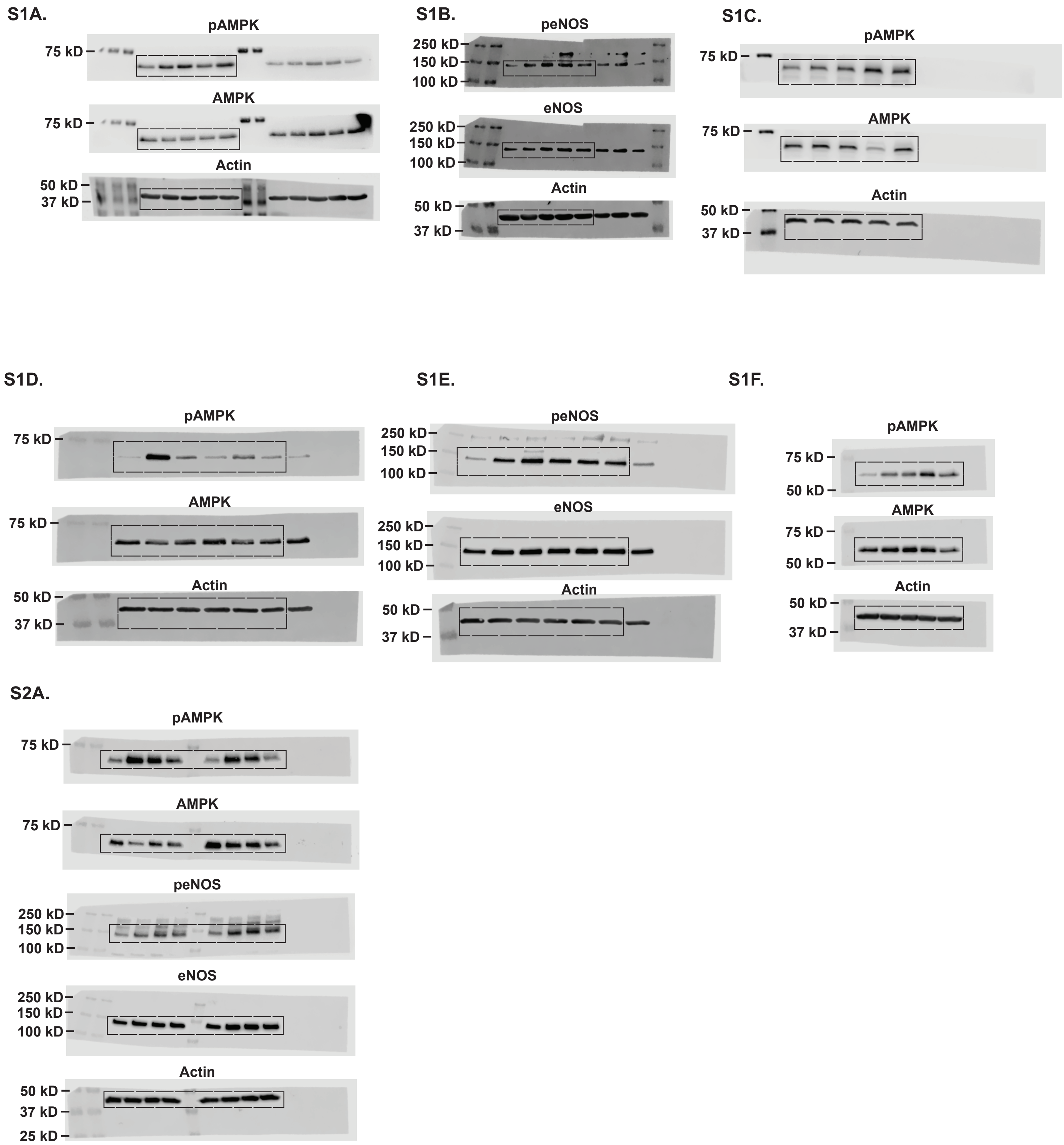

Fig. S6. Uncropped Blots for Fig S1, S2, S3: The uncropped blots are shown, and include channels that identify the ladder. The dotted region indicates the portion of the blot included in the figure.
