# Supplementary material for: Shear stress-stimulated AMPK couples endothelial cell mechanics, metabolism and vasodilation
Source: J Cell Sci. 2024 Dec 18;137(24):jcs262232. doi: 10.1242/jcs.262232 (PMC11795286; doi:10.1242/jcs.262232)
Supplement: Supplementary file 1 [file joces-137-262232_review_history.pdf]

Shear stress-stimulated AMPK couples endothelial cell mechanics, metabolism, and vasodilation

Nicholas M. Cronin, Logan W. Dawson and Kris A. DeMali

DOI: 10.1242/jcs.262232

Editor: Andrew Ewald

Review timeline

Original submission:	29 April 2024
Editorial decision:	3 June 2024
First revision received:	18 September 2024
Editorial decision:	8 October 2024
Second revision received:	24 October 2024
Accepted:	2 November 2024

Original submission

First decision letter

MS ID#: JOCES/2024/262232

MS TITLE: Mechanical activation of VE-cadherin stimulates AMPK to increase endothelial cell metabolism and vasodilation

AUTHORS: Nicholas M Cronin, Logan W Dawson, and Kris A DeMali

We have now reached a decision on the above manuscript.

To see the reviewers' reports and a copy of this decision letter, please go to: https://submit-jcs.biologists.org and click on the 'Manuscripts with Decisions' queue in the Author Area. (Corresponding author only has access to reviews.)

As you will see, the reviewers share enthusiasm for the study but raise a number of substantial criticisms that prevent me from accepting the paper at this stage. They suggest, however, that a revised version might prove acceptable, if you can address their concerns. If you think that you can deal satisfactorily with the criticisms on revision, I would be pleased to see a revised manuscript. We would then return it to the reviewers. Please let me know if you would like to discuss a revision plan.

Please ensure that you clearly highlight all changes made in the revised manuscript. Please avoid using 'Tracked changes' in Word files as these are lost in PDF conversion.

I should be grateful if you would also provide a point-by-point response detailing how you have dealt with the points raised by the reviewers in the 'Response to Reviewers' box. Please attend to all of the reviewers' comments. If you do not agree with any of their criticisms or suggestions please explain clearly why this is so.

Reviewer 1

Advance summary and potential significance to field

© 2024. Published by The Company of Biologists under the terms of the Creative Commons Attribution License (https://creativecommons.org/licenses/by/4.0/).

This is a manuscript by Cronin, Dawson and DeMali regarding increases in endothelial cell metabolism following exposure to fluid shear stress.
This work follows prior work by the DeMali group showing that there is a relationship between mechanical force and metabolism. This is a very interesting area of research, and the current manuscript continues to provide mechanistic insight into how this process occurs. I am very excited by this work and believe it is appropriate for JCS.

Comments for the author

Major concerns:
1. In the results section for Figure 2 mentions 3 kinases that can activate AMPK: LKB1, CaMKKß, and TAK1. TAK1 does not appear to be tested, and this is confusing to have it mentioned but not examined. The data demonstrate LKB1 is clearly important, but it doesn't completely eliminate its activation by shear stress (Figure 2C and 2D). The statistics show that it is not different between static and shear, but the representative blot indicates otherwise (there is a clear increase in pAMPK in the blot). Also the CaMKKß inhibitor doesn't completely ablate the shear stress-induced increase in pAMPK. In both of these cases there is only an n=3 and the blots are likely somewhat variable. Perhaps the authors need to increase the number of replicates to truly know if it something is or isn't different? I agree the LKB1 has the bigger effect.
2. While I am very excited by this work, I am a bit hesitant about the conclusion that it is mediated by VE-cadherin force transmission. The conclusion is based on 2 approaches: 1) blocking VE-cadherin adhesion (or silencing VE-cadherin) and 2) myosin inhibition. Myosin inhibition is quite blunt and likely doing many things besides modulating VE-cadherin force. In the case of blocking or silencing VE-cadherin, there could also be effects of VE-cadherin as a scaffold to initiate the signaling, as well as a significant change in how well the cells are adhered to one another. The idea VE-cadherin could serve as a scaffold is not a hypothetical, as it is established that VE-cadherin/VEGFR2/PECAM need to interact to initiate signaling in response to shear. Clearly VE-cadherin does something to activate AMPK, but it is not necessarily due to force on VE-cadherin as argued in the manuscript (including the title). Furthermore, VE-cadherin (as well as total cell-cell and cell-ECM force) is thought to decrease in force at the onset of shear stress (see Conway Current Biology 2013)--thus the claim that shear stress is applying force to VE-cadherin may be tenuous in light of no data in this manuscript showing VE-cadherin forces are increasing (and the prior data by the Schwartz group that shows a force decrease). I would suggest the authors soften this conclusion to something about VE-cadherin being necessary to activate AMPK and remove the reference to force on VE-cadherin. To truly test this idea the authors would need to come up with an experiment that blocks VE-cadherin force changes by shear, which may be challenging and beyond the scope of this paper.
Alternatively magnetic bead pulling or some other approach to apply force to VE-cadherin could be used--but it may not be the same type of force as shear stress.
3. The 48 hr alignment data are compelling, but these are strong treatments (inhibiting VE-cadherin, inhibiting myosin, blocking glucose transport) that likely affect other important signaling pathways. Did the authors look at how silencing of AMPK or LKBP affect alignment? These experiments are more subtle & could help strengthen the claims that this pathway is necessary for mechanoadapation to shear stress.

Minor:
1. Figure 1, 2, and 3: can the timepoints of shear be clarified? I assume other than the first panel these are all at 5 minutes, but this is not stated.
2. In Figure 3E (wild-type) why is the enos so brighter (everywhere, not just at junctions) at the onset of shear? Is this p-enos? If total enos, it seems unlikely the protein expression could change so quickly.
3. Figure 6A: 3PO treatment results in noticeably reduced cell density. Does this mean this treatment may be having other effects (or toxicity) beyond what is being studied in this paper?
4. Figure 2E which band is supposed to be LKB1? I assume the top band but this should be clearly indicated.

<u>Reviewer 2</u>

© 2024. Published by The Company of Biologists under the terms of the Creative Commons Attribution License (https://creativecommons.org/licenses/by/4.0/).

Advance summary and potential significance to field

In this manuscript, Cronin and colleagues characterize a pathway activated in response shear stress through VE-cadherin. The results show that WE-cadherin recruits the kinase LKB1 that in turn activates AMPK a key enzyme in homeostasis regulation. The activation of AMPK controls eNOS, which suggests it may be involved in vasodilation, as well as cytoskeleton remodeling and glucose uptake. The pathway is almost identical to the one described by the authors in 2017 downstream of E-cadherin (DOI: 10.1038/ncb3537), so the results are not totally unexpected. There are new findings, in particular those related to the activation of eNOS. However, as mentioned in the paper, both the activation of AMPK and eNOS in response to shear stress have been previously reported in endothelial cells. The merit of the authors here is to tie all the pieces together. The experimental design is clear and the results are sound. I think the study is important, as it confirms the existence of the pathway in endothelial cells. However, I believe the interpretation of the results needs to be recalibrated and that the novelty is moderate.

Comments for the author

Major concerns:
-The main concern I have is regarding the interpretation of the results in general. The authors use different strategies to perturb mechanosensing, including a VE-cadherin blocking antibody and blebbistatin for example. In the text, they interpret the results from these perturbations as an effect of interfering with VE-cadherin force transduction. However, blocking antibodies will disrupt cell-cell junctions, a much more drastic effect that just impairing VE-cadherin force sensing. Similarly, blebbistatin treatment disrupts force sensing from different adhesion structures, including cell junctions, but also focal adhesions. This makes it difficult to attribute the effects exclusively to VE-cadherin mediated force transduction. People have addressed these issues using VE-cadherin mutants that are impaired in force sensing, such as vinculin binding deficient mutants for example.
There are two potential options to address this issue. First, directly test the role of VE-cadherin force transduction using mutants (or other appropriate approaches), or, second, tone down the interpretation of the results, acknowledging the limitations of the study. Of course, the second alternative will impact the significance of the study.
-The proper CTRL for Fig 3E should be non-targeting shRNA (as in Fig. 4)
-Fig 3E-F and 4A-B show virtually the same results. Figs. 3E-G and 4A-C can be easily combined. As they are there is a lot of redundancy.
-Are eNOS total levels increased with shear stress? The results show a significant increase in eNOS total levels even at 5 min of stress, which seems unlikely (3E and Also 5C).

Minor comments:
-Figure 3E and 4A and 5C show the same CTRL (DMSO), staining VE-cadherin with or without stress. The results, however, are quite different, in particular those in 3E which seem to show a much more drastic increase in VE-cadherin.
-No scale bars in Figure 4A
-Figure 4C Y axis should be labeled “actin” or “F-actin”, not VE-cadherin.
-“Figure 5” is missing in the legend for Fig. 5
-I would recommend the use of a color-blind appropriate scheme for the IF images.
-Source and sequence of the shScramble sequence used is missing
-More detail on image acquisition would be useful for the reader. For example, are the images shown one Z-plane?, a max projection? How was intensity quantified? Again, on one z-plane or a sum projection?
-Figure legend states that quantification of junctions was performed on 100 junctions over 2-3 fields. It is not clear if the experiments were performed in triplicate.
-In the methods, if I understand correctly, it says that the IF experiments were not quantified on independent experiments (but were repeated with similar results). If that is the case, how was the N number calculated? Is N in the quantification equal to the 100 junctions measured?

© 2024. Published by The Company of Biologists under the terms of the Creative Commons Attribution License (https://creativecommons.org/licenses/by/4.0/).

First revision

Author response to reviewers' comments

We are pleased that the reviewers are excited by this work (reviewer 1) and believe it to be important (Reviewer 2) and appropriate for JCS (Reviewer 1).
Reviewer 1 Comments for the Author:
Major concerns:
1. In the results section for Figure 2 mentions 3 kinases that can activate AMPK: LKB1, CaMKKß, and TAK1. TAK1 does not appear to be tested, and this is confusing to have it mentioned but not examined. We removed the reference to TAK1 as it remains speculative that TAK1 is a bonafide AMPK kinase (ref: PMC6121279). The data demonstrate LKB1 is clearly important, but it doesn't eliminate its activation by shear stress (Figure 2C and 2D). The statistics show that it is not different between static and shear, but the representative blot indicates otherwise (there is a clear increase in pAMPK in the blot). Also the CaMKKß inhibitor doesn't completely ablate the shear stress-induced increase in pAMPK. In both of these cases there is only an n=3 and the blots are likely somewhat variable. Perhaps the authors need to increase the number of replicates to truly know if it something is or isn't different? I agree the LKB1 has the bigger effect. The shLKB1 cells employed in these studies are not a complete knockout. Rather, as Figure 2A demonstrates, the knockdown is 66% effective in inhibiting LKB1 expression. Thus, it is not surprising that this level of knockdown does not completely rid the cell shear-induced pAMPK. It is also worth noting that the observation that LKB1 is the critical protein is supported by other pieces of data showing that its inhibition affects AMPK-mediated events. Thus. more n's might make the data prettier (but not necessarily representative) and, importantly, will not change the result, especially since there is statistical significance is achieved in the presence of experimental variability.

2. While I am very excited by this work, I am a bit hesitant about the conclusion that it is mediated by VE-cadherin force transmission. The conclusion is based on 2 approaches: 1) blocking VE-cadherin adhesion (or silencing VE-cadherin) and 2) myosin inhibition. Myosin inhibition is quite blunt and likely doing many things besides modulating VE-cadherin force.
In the case of blocking or silencing VE-cadherin, there could also be effects of VE-cadherin as a scaffold to initiate the signaling, as well as a significant change in how well the cells are adhered to one another. The idea VE-cadherin could serve as a scaffold is not a hypothetical, as it is established that VE-cadherin/VEGFR2/PECAM need to interact to initiate signaling in response to shear. Clearly VE-cadherin does something to activate AMPK, but it is not necessarily due to force on VE-cadherin as argued in the manuscript (including the title). Furthermore, VE-cadherin (as well as total cell-cell and cell-ECM force) is thought to decrease in force at the onset of shear stress (see Conway Current Biology 2013)--thus the claim that shear stress is applying force to VE-cadherin may be tenuous in light of no data in this manuscript showing VE- cadherin forces are increasing (and the prior data by the Schwartz group that shows a force decrease). I would suggest the authors soften this conclusion to something about VE-cadherin being necessary to activate AMPK and remove the reference to force on VE-cadherin. To truly test this idea the authors would need to come up with an experiment that blocks VE-cadherin force changes by shear, which may be challenging and beyond the scope of this paper. Alternatively magnetic bead pulling or some other approach to apply force to VE-cadherin could be used--but it may not be the same type of force as shear stress. This is a fair assertion and one noted by both reviewers. We did not mean to state as strongly as we did that it was VE-cadherin force transmission. In the revised manuscript, we have modified the text and title to tone down the link between VE-cadherin and force. Instead, it now reflects a requirement for VE-cadherin, but not VE-cadherin force transmission.

3. The 48 hr alignment data are compelling, but these are strong treatments (inhibiting VE-cadherin, inhibiting myosin, blocking glucose transport) that likely affect other important signaling pathways. Did the authors look at how silencing of AMPK or LKBP affect alignment? These experiments are more subtle & could help strengthen the claims that this pathway is necessary for mechanoadapation to shear stress. Yes, we examined the inhibition of LKB1 and observed decreased alignment. We included this data in the revised manuscript Figure 6. Inhibition of AMPK has proven more difficult. We silenced AMPK alpha 1 and the cells still aligned, but these cells still have AMPK alpha 2. Inhibition of both isoforms is not possible because inhibition of both isoforms causes cell death.
Minor:

© 2024. Published by The Company of Biologists under the terms of the Creative Commons Attribution License (https://creativecommons.org/licenses/by/4.0/).

1. Figure 1, 2, and 3: can the timepoints of shear be clarified? I assume other than the first panel these are all at 5 minutes, but this is not stated. They were not all performed at five minutes. The length of shear application is now noted in the legends and materials methods.
2. In Figure 3E (wild-type) why is the enos so brighter (everywhere, not just at junctions) at the onset of shear? Is this p- enos? If total enos, it seems unlikely the protein expression could change so quickly. We went back and examined our data, and the image we chose for the original submission was not as representative as it could have been. We have replaced the wildtype shear figure panels in Figure 3E. Also, we routinely examined eNOS levels via immunoblotting and have not observed changes in total eNOS levels. We added this data and a quantification of it as Supplemental Figure 3E.

3. Figure 6A: 3PO treatment results in noticeably reduced cell density. Does this mean this treatment may be having other effects (or toxicity) beyond what is being studied in this paper? 3PO treatment does tend to decrease cell growth. In the revised manuscript, we plated the cells at a higher density and examined them with the same treatment time.
Under these conditions, we still observe 3PO inhibiting alignment. This data is shown in Supplemental Figure 4.

4. Figure 2E which band is supposed to be LKB1? The reviewer raises a good point, and we are fortunate that this ambiguity was called to our attention. To determine which band was LKB1, we re-ran the experiment and, this time included cell lysates with and without LKB1 inhibited as a control. From this analysis, it is now evident which band is LKB1.

*****

Reviewer 2 Advance Summary and Potential Significance to Field:
In this manuscript, Cronin and colleagues characterize a pathway activated in response shear stress through VE- cadherin. The results show that WE-cadherin recruits the kinase LKB1 that in turn activates AMPK a key enzyme in homeostasis regulation. The activation of AMPK controls eNOS, which suggests it may be involved in vasodilation, as well as cytoskeleton remodeling and glucose uptake. The pathway is almost identical to the one described by the authors in 2017 downstream of E-cadherin (DOI: 10.1038/ncb3537), so the results are not totally unexpected. There are new findings, in particular those related to the activation of eNOS. However, as mentioned in the paper, both the activation of AMPK and eNOS in response to shear stress have been previously reported in endothelial cells. The merit of the authors here is to tie all the pieces together. The experimental design is clear and the results are sound. I think the study is important, as it confirms the existence of the pathway in endothelial cells. However, I believe the interpretation of the results needs to be recalibrated and that the novelty is moderate.
Reviewer 2 Comments for the Author:
Major concerns:
-The main concern I have is regarding the interpretation of the results in general. The authors use different strategies to perturb mechanosensing, including a VE-cadherin blocking antibody and blebbistatin for example. In the text, they interpret the results from these perturbations as an effect of interfering with VE-cadherin force transduction. However, blocking antibodies will disrupt cell-cell junctions, a much more drastic effect that just impairing VE-cadherin force sensing. Similarly, blebbistatin treatment disrupts force sensing from different adhesion structures, including cell junctions, but also focal adhesions. This makes it difficult to attribute the effects exclusively to VE-cadherin mediated force transduction. People have addressed these issues using VE-cadherin mutants that are impaired in force sensing, such as vinculin binding deficient mutants for example. There are two potential options to address this issue. First, directly test the role of VE-cadherin force transduction using mutants (or other appropriate approaches), or, second, tone down the interpretation of the results, acknowledging the limitations of the study. Of course, the second alternative will impact the significance of the study. As we noted above, we did not intend to state as strongly as we did that VE- cadherin was a force transducer. As noted by reviewer #1, there is no clear way to mutate VE-cadherin that allows its role in cell-cell adhesion, force transmission, or signaling to be distinguished. For example, there are mutants of VE-cadherin that disrupt signaling, but they also disrupt cell-cell adhesion, and there are mutants that disrupt homophilic binding, but they disrupt signaling, etc. Thus, we don't believe it is possible to definitively distinguish between a signaling role and a force transmission role. Instead, and upon the urging of both reviewers, we have toned down the conclusions to state a requirement for VE-cadherin but not VE-cadherin force transmission.

© 2024. Published by The Company of Biologists under the terms of the Creative Commons Attribution License (https://creativecommons.org/licenses/by/4.0/).

-The proper CTRL for Fig 3E should be non-targeting shRNA (as in Fig. 4) Yes, we agree. We have added the requested scramble in Supplemental Figure 3E.
-Fig 3E-F and 4A-B show virtually the same results. Figs. 3E-G and 4A-C can be easily combined. As they are there is a lot of redundancy. It is true that the actin panels could be added to Figure 3E. We didn't do that initially because we find when we present the data in this manner there is lots of confusion among readers/audience members when too much information is presented in the same figure. Also, the figures address very different points. With all that having been said, we appreciate the reviewer's concern and have presented all three pieces of data together later in the manuscript to save space.
-Are eNOS total levels increased with shear stress? The results show a significant increase in eNOS total levels even at 5 min of stress, which seems unlikely (3E and Also 5C). Reviewer 1 noted this concern as well. As mentioned above, we don't believe that the image in 3E was as representative as we hoped, and we have replaced it. We also went back and quantified the levels of cytoplasmic eNOS in our immunofluorescence images and found no significant differences in the presence or absence of shear stress. Similarly, the total levels of eNOS were examined by immunoblotting. We have not observed statistically significant differences in non-junction total eNOS. These data are included in the revised manuscript as supplemental figures 3C-F.
Minor comments:
-Figure 3E and 4A and 5C show the same CTRL (DMSO), staining VE-cadherin with or without stress. The results, however, are quite different, in particular those in 3E which seem to show a much more drastic increase in VE-cadherin.
-No scale bars in Figure 4A. Thanks for pointing out the omission--we have added a scale bar.
-Figure 4C Y axis should be labeled "actin" or "F-actin", not VE-cadherin. Thank you. We have made the change.
-"Figure 5" is missing in the legend for Fig. 5. We fixed this error too.
-I would recommend the use of a color-blind appropriate scheme for the IF images. It does not appear that JCS has color- blind appropriate guidelines. The American Society for Cell Biology website indicates that for images to be accessible to color blind individuals, they should use colors green with magenta or red and yellow. They also note that the best practice is to show grayscale images. We have changed the color scheme for our images to adhere to these recommendations.
-Source and sequence of the shScramble sequence used is missing. We have added the requested information.
-More detail on image acquisition would be useful for the reader. For example, are the images shown one Z-plane?, a max projection? How was intensity quantified? Again, on one z-plane or a sum projection? More detail has been added to the materials and methods.
-Figure legend states that quantification of junctions was performed on 100 junctions over 2-3 fields. It is not clear if the experiments were performed in triplicate. The experiments were performed in triplicate. The quantification was done from one experiment. We have updated the methods for clarity.
-In the methods, if I understand correctly, it says that the IF experiments were not quantified on independent experiments (but were repeated with similar results). If that is the case, how was the N number calculated? Is N in the quantification equal to the 100 junctions measured? The quantification was done using 100 different junctions in one experiment.

Second decision letter

MS ID#: JOCES/2024/262232

MS TITLE: Shear stress-stimulated AMPK couples endothelial cell mechanics, metabolism, and vasodilation

AUTHORS: Nicholas M Cronin, Logan W Dawson, and Kris A DeMali

We have now reached a decision on the above manuscript.

© 2024. Published by The Company of Biologists under the terms of the Creative Commons Attribution License (https://creativecommons.org/licenses/by/4.0/).

To see the reviewers' reports and a copy of this decision letter, please go to: https://submit-jcs.biologists.org and click on the 'Manuscripts with Decisions' queue in the Author Area. (Corresponding author only has access to reviews.)

As you will see, the reviewers gave favourable reports and there is only one issue remaining from Reviewer 2, related to the statistical analysis of the junctions. Please read, consider, and reply with a plan. That might involve text edits or a variation on the statistical analysis. The Reviewer is raising a question and leaving significant discretion as to the best solution. I hope that you will be able to carry this out because I would like to be able to accept your paper. Depending on your explanation, I may be able to make a decision without consulting the reviewers further.

Please ensure that you clearly highlight all changes made in the revised manuscript. Please avoid using 'Tracked changes' in Word files as these are lost in PDF conversion.

I should be grateful if you would also provide a point-by-point response detailing how you have dealt with the points raised by the reviewers in the 'Response to Reviewers' box. Please attend to all of the reviewers' comments. If you do not agree with any of their criticisms or suggestions please explain clearly why this is so.

Reviewer 1

Advance summary and potential significance to field

This work is novel for identifying how shear stress activates kinases that affect cell metabolism, tying mechanotransduction to metabolism.

Comments for the author

The authors have fully addressed all of my prior concerns. I have no additional concerns.

Reviewer 2

Advance summary and potential significance to field

In this manuscript, Cronin and colleagues characterize a pathway activated in response shear stress through VE-cadherin. The results show that VE-cadherin recruits the kinase LKB1 that in turn activates AMPK a key enzyme in homeostasis regulation. The activation of AMPK controls eNOS, which suggests it may be involved in vasodilation, as well as cytoskeleton remodeling and glucose uptake. The pathway is almost identical to the one described by the authors in 2017 downstream of E-cadherin (DOI: 10.1038/ncb3537), so the results are not totally unexpected. There are new findings, in particular those related to the activation of eNOS. However, as mentioned in the paper, both the activation of AMPK and eNOS in response to shear stress have been previously reported in endothelial cells. The merit of the authors here is to tie all the pieces together. The experimental design is clear and the results are sound. I think the study is important, as it confirms the existence of the pathway in endothelial cells.

Comments for the author

The authors have addressed all my concerns and I believe the article is suitable for publication in Journal of Cell Science.
I have one minor concern/comment on the statistical analysis of some of the datasets included in the figures. This applies mostly to the analysis of junctional intensity, shown in Figs 3,4 and 5 and in Supp Fig 3, and also to the quantification of cytoplasmic eNOS, also in Supp Fig 3. These datasets were collected from a single experiment, typically from 2-3 fields of view. I believe it is not appropriate to apply statistical analysis, as is done on the article, for experiments that have not been repeated. The authors define N as the numbers of measurements (usually 100), which is why the statistical analyses show extremely low p-values.

© 2024. Published by The Company of Biologists under the terms of the Creative Commons Attribution License (https://creativecommons.org/licenses/by/4.0/).

In my opinion, the N should reflect the number of experiments, ideally 3. Or at minimum, the authors should have averaged the result for each field of view (technical replicate) and then average the means and use N=3 for the statistical analysis. I don't believe this changes any of the results or interpretation. Certainly the p-values would be much different. I may be wrong here, and happy to be corrected. I just wanted to comment on this.

Second revision

Author response to reviewers' comments

Reviewer #2 had raised concerns about the statistical analysis used for the quantification of our immunofluorescence data. We agree that transparency in the spread across the biologically independent experiments should be clearly illustrated in this work.

To address this concern, we quantified 3 biologically independent experiments. The data point of each experiment is the average value acquired from 2-3 fields of view (FOV) of which >100 junctions were measured. The statistical analysis was performed on the normalized data set where n=3 biologically independent experiments.

Third decision letter

MS ID#: JOCES/2024/262232

MS TITLE: Shear stress-stimulated AMPK couples endothelial cell mechanics, metabolism, and vasodilation

AUTHORS: Nicholas M Cronin, Logan W Dawson, and Kris A DeMali

I am happy to tell you that your manuscript has been accepted for publication in Journal of Cell Science, pending standard publication integrity checks.

© 2024. Published by The Company of Biologists under the terms of the Creative Commons Attribution License (https://creativecommons.org/licenses/by/4.0/).
